# Phylogenetically and metabolically diverse active carbon-fixing microbes reside in mangrove sediments

**DOI:** 10.1186/s40168-025-02177-9

**Published:** 2025-09-01

**Authors:** Shasha Wang, Zhuoming Zhao, Ruolin Cheng, Liang Cui, Jun Wang, Maxim Rubin-Blum, Yao Zhang, Bolin Liu, Xing Chen, Federico Baltar, Xiaxing Cao, Xuezhe Wen, Karine Alain, Zhen Chen, Jing Liao, Lijing Jiang, Zongze Shao

**Affiliations:** 1 Key Laboratory of Marine Genetic Resources, Third Institute of Oceanography, Ministry of Natural Resources of People’s Republic of China, Xiamen, 361005 China; 2 Biology Department, National Institute of Oceanography, Israel Oceanographic and Limnological Research (IOLR), 3108000 Haifa, Israel; 3 State Key Laboratory of Marine Environmental Sciences, College of Ocean and Earth Sciences, Xiamen University, Xiamen, 361101 China; 4 State Key Laboratory of Estuarine and Coastal Research, East China Normal University, Shanghai, 200241 China; 5 Frontiers Science Center for Deep Ocean Multispheres and Earth System, College of Marine Life Sciences, Ocean University of China, Qingdao, 266003 China; 6 Fungal and Biogeochemical Oceanography Group, College of Oceanography and Ecological Science, Shanghai Ocean University, Shanghai, 201306 China; 7 Univ Brest, CNRS, Biologie Interactions Et Adaptations Des Organismes en Milieu EXtrême, IRP 1211 MicrobSea, EMR6002 BIOMEXF-29280 Plouzané, Ifremer France; 8 Fujian Ocean Innovation Center, Xiamen, 361102 China

**Keywords:** Mangrove sediments, Dark carbon fixation, Chemolithoautotroph, Vertical distribution, In situ, Metatranscriptomics

## Abstract

**Background:**

Mangroves are hotspots of carbon sequestration in transitional zones between marine and terrestrial ecosystems. Microbially driven dark carbon fixation (DCF) is prominent in sediments, yet our understanding of the DCF process across this continuum remains limited. In this study, we explored DCF activities and associated chemoautotrophs along the sediment depth of different mangrove sites in Fujian Province, China, using radiocarbon labeling and molecular techniques.

**Results:**

Our results showed that the DCF rates ranged from 0.02 to 3.27 mmol C m^−2^ day^−1^ in all samples, showing a depth-dependent spatial variation. These rates of DCF were closely related to the environmental factors such as DIC, TS, AVS, NH_4_^+^, NO_3_^−^, and NO_2_^−^. Metagenomic analysis revealed six carbon-fixing pathways, with the Calvin-Benson-Bassham (CBB) cycle and Wood-Ljungdahl (WL) pathway being predominant. Further analysis of MAGs revealed that *Gammaproteobacteria*, *Desulfobacteria*, and *Campylobacteria* were the most abundant carbon-fixing groups. Intriguingly, some new lineages were found to have carbon-fixing potential, including two candidatus taxa JAJVIF01 and BMS3Abin14. Metatranscriptomic analyses confirmed that these carbon-fixing microbes were active in situ and occupied different niches. In the surface layers, *Gammaproteobacteria* with the CBB cycle played an important role in DCF, mainly driven by sulfur and hydrogen oxidation with oxygen reduction; in the deeper layers, *Campylobacteria* with the reductive tricarboxylic acid (rTCA) cycle and *Desulfobacteria* with the WL pathway were active members for DCF, mainly through sulfur, hydrogen, and CO oxidation. While in the deepest layers of 18–20 cm, methane-producing archaea *Methanosarcinia* was the essential member driving DCF. In addition, most taxa containing the WL pathway displayed a mixotrophic lifestyle corresponding to flexible carbon acquisition strategies.

**Conclusions:**

Overall, this study provides new insights into the understanding of biological carbon fixation and its ecological functions in mangrove sediments.

Video Abstract

**Supplementary Information:**

The online version contains supplementary material available at 10.1186/s40168-025-02177-9.

## Introduction

Mangrove wetlands, as one of the most productive “blue carbon” ecosystems located in coastal regions, can function as an efficient long-term natural carbon sink [1, 2]. Although mangroves occupy only 0.5% of the global coastal area, they contribute 10–15% (24 Tg C year^−1^) to coastal sediment carbon storage and export 10–11% of the particulate terrestrial carbon to the ocean [2]. Mangrove sediments account for approximately 70% of the carbon storage in the entire ecosystem, and it is assumed that most of the below-ground carbon storage in mangroves comes from the burial of their own organic carbon and the input of exogenous organic matter [3, 4]. However, the contribution of microbial carbon fixation to sediment carbon storage cannot be ignored, given that 291 Tg C year^−1^ carbon fixation amounts in coastal sediments are comparable to the total organic carbon (TOC) inputs from rivers to the coastal ocean (317–450 Tg C year^−1^) [5]. Early measurements also indicated that the average carbon fixation rates in China’s mangrove sediments are approximately 4.44 Mg C ha^−1^ year^−1^, which is higher than the global average level of 2.10 Mg C ha^−1^ year^−1^ for carbon fixation in mangrove soils [6]. In addition, previous studies generally accepted that microbes in mangrove sediments played an important role in the degradation of organic matter, mainly contributing to carbon emissions [7]. However, mangrove sediments are the hotspots for highly active redox reactions, providing ample electron donors and acceptors for chemoautotrophy [8]. Therefore, to understand how inorganic carbon at mangrove sediments is turned over and possibly buried, detailed knowledge of the microbes driving these processes is essential but currently still lacking.

Chemoautotrophy in sediment ecosystems is mainly supported by the re-oxidation of reduced compounds generated during anaerobic mineralization processes [9]. Chemoautotrophic microorganisms obtain their metabolic energy by the oxidation of various reduced inorganic substrates, such as hydrogen, ammonium, nitrite, ferrous iron, or sulfide, to synthesize organic molecules, and this process is referred to as dark carbon fixation (DCF) [10]. In mangrove sediments, sulfate reduction is the main respiration pathway, accounting for about 50 to 70% of the organic matter mineralization [11], and produces a large amount of inorganic reduced sulfur compounds, which in turn are an important driver for chemoautotrophic carbon fixation [12]. In addition to sulfur species, the oxidation of ammonium, carbon monoxide (CO), H_2_, or metals can also be important energy sources for carbon sequestration by chemoautotrophs in mangrove sediments, with oxygen, sulfate, nitrate, and Fe (III) as the major electron acceptors [13, 14]. Moreover, due to the influence of periodic exposure and inundation under tidal action, mangrove sediments exhibit extremely active redox reactions. These reductive and oxidative processes tightly link the cycles of sediment carbon, nitrogen, sulfur, hydrogen, and metals, and provide natural advantage for chemoautotrophy [15].

In recent years, chemolithoautotrophs in organic-rich sediment ecosystems have received attention, in addition to the extensively studied oligotrophic habitats such as oxygen minimum zones (OMZs) and hydrothermal vents [16, 17]. More studies have revealed that some putative chemolithoautotrophic taxa are prevalent within different mangrove sediment ecosystems by the 16S rRNA gene amplicons and metagenomics [15]. For example, *Sulfurovum*, *Sulfurimonas*, *Thermodesulfovibrio*, *Desulfococcus*, and *Desulfobacterium* lineages were predominant with a relative abundance > 1% in Yunxiao mangrove sediments [18]. *Desulfococcus*, *Nitrosopumilus*, and *Sulfurimonas* were also dominant members in mangrove sediments collected from six locations along the coastline of BeibuGulf in Guangxi Province, China [19]. The prevalence of these lineages suggests the important functional role of these putative chemolithoautotrophs in mangrove sediments. Recently, our findings by activity measures, metagenomics, and metatranscriptomics demonstrated that nitrogen fixation was dominated by chemolithoautotrophs rather than heterotrophs in mangrove sediments [20], indicating that chemolithoautotrophs may be more important than we previously thought in such carbon-rich habitats. However, little is known about the diversity, composition, and activity of chemoautotrophs in mangrove sediments, especially their ecological functions in driving DCF.

Currently, seven pathways of CO_2_ fixation have been described in natural environments [21]. Calvin-Benson-Bassham (CBB) cycle and reductive tricarboxylic acid (rTCA) cycle are thought to be the most significant in coastal sediments [22, 23], while recent studies have also highlighted the important role of the Wood-Ljungdahl (WL) pathway in anoxic environments [24]. The CBB cycle and its key enzyme (RuBisCO, *cbbLS*/*cbbM* genes encoding forms I and II) are widely distributed under aerobic conditions with high endurance of its enzymes to oxygen [25]. The oxygen-sensitive rTCA cycle (key enzyme: ATP-dependent citrate lyase, Acl) can operate under microaerobic conditions in some organisms, based on often poorly understood adaptations [26]. The WL pathway requires strictly anaerobic conditions since its key enzyme, acetyl-CoA synthase (Acs), is highly sensitive to oxygen [26]. Alternative carbon fixation strategies include the dicarboxylate/4-hydroxybutyrate (DC/4HB) cycle [27], the 3-hydroxypropionate/4-hydroxybutyrate (3HP/4HB) cycle (mainly in *Thermoproteota*), and the 3-hydroxypropionate (3HP) bicycle (*Chloroflexota*) [28]. Additionally, the reductive glycine (rGly) pathway is newly discovered but difficult to make bioinformatical predictions [29].

The present study attempted to elucidate the ecological importance and underlying mechanisms of the DCF in mangrove sediments. For that, we (i) determined the spatial distribution of DCF rates and functional gene abundance along the sediment depth; (ii) explored potential links between environmental variables and DCF activity; and (iii) identified active microbes that can fix carbon in mangrove sediments using metagenomics and metatranscriptomics. This study first highlights the importance of chemoautotrophic microbes in carbon-rich mangrove sediment ecosystems, rather than in the well-studied oligotrophic habitats, expanding our understanding of microbial-mediated carbon cycle in the global oceans.

## Materials and methods

### Sample collection and physicochemical properties analysis

The sampling sites were located in three mangrove wetlands of Fujian Province, China, including Jiulong River estuary (117° 45′ E, 24° 20′ N), Quanzhou Bay (118° 42′ E, 24° 56′ N), and Zhangjiang estuary (117° 24′ E, 23° 55′ N) (Fig. S1). Jiulong River estuary and Quanzhou bay estuary are provincial-level nature reserves, with an estimated area of mangrove forests of 86.7 hm^2^ and 568.5 hm^2^, respectively [30]. Zhangjiang estuary is the most well-preserved mangrove forest in China, and is a national-level nature reserve with a tidal flat area of 274.1 hm^2^ [31]. Sediment cores were collected at each site in triplicate using a 20/22-cm long PVC sampling column after ebb, and sliced at 2-cm intervals into 10 layers at the first two sites and 11 layers at the latter site in August and December 2023, yielding a total of 93 samples. Sliced sediments were stored in a portable cooler at 4℃ and transported back to the laboratory within 2 h. Ninety-three samples were stored at 4℃ for physicochemical analysis and DCF rate measurement, and 31 samples from one sediment core at each site were selected as representatives and kept at − 80℃ for DNA or RNA extraction. The environmental variables, including the total carbon (TC), total nitrogen (TN), total sulfur (TS), total organic carbon (TOC), dissolved inorganic carbon (DIC), ammonium (NH_4_^+^), nitrate (NO_3_^−^), nitrite (NO_2_^−^), acid volatile sulfide (AVS), sulfate (SO_4_^2−^), pH, moisture, salinity, and redox potential, were determined as described previously [20].

### Dark carbon fixation rates

DCF rates were measured through the assimilation of NaH^14^CO_3_ tracer (58.0 mCi mmol^−1^, PerkinElmer, USA) [32, 33]. In brief, sediment slurries were made by mixing fresh sediment and sterile filtered (0.22 μm) overlying water at a ratio of 1:1 (wt/vol) in an anaerobic chamber. One milliliter of slurry along with a formaldehyde-killed control was incubated in triplicate in darkness at the in situ temperature following the addition of 3 μCi of NaH^14^CO_3_ tracer. After 24 h, incorporation of DIC was terminated by adding 2% formaldehyde. Dead controls were made for each sample by adding formaldehyde immediately before the addition of a ^14^C tracer. After acidification with HCl, sediment TOC was extracted using Ultima Gold scintillation cocktail, and the ^14^C radioactivity was quantified using a liquid scintillation counter (300SL, Hidex, USA). More details on DCF measurements and calculations are given in Supplementary Methods.

### DNA extraction and quantitative PCR analysis

DNA was extracted from 0.25 g of collected sediments using a DNeasy PowerMax Soil Kit (12,988–10, QIAGEN, Germany) according to the manufacturer’s instructions. qPCR analyses were performed to estimate the total prokaryotic microbial abundance of 31 samples. The details of these procedures are described in Supplementary Methods.

### 16S rRNA gene amplicon sequencing and community analysis

Microbial community compositions of sediment samples were assessed using high-throughput sequencing of the V3–V4 region of bacterial 16S rRNA genes and the V4–V5 region of archaeal 16S rRNA genes, using the universal primers 338F-806R and 524F10extF-Arch958RmodR, respectively [34]. Reads were quality controlled and then clustered into amplicon sequence variants (ASVs) of > 99% sequence identity with a 70% confidence threshold. Taxonomic assignments were assigned together with the SILVA v138 database using the QIIME2 plugin. A detailed description is provided in Supplementary Methods.

### Metagenomic sequencing, assembly, and binning

DNA was extracted from the sediment samples as described above. DNA sequencing and metagenomic library preparation of the above 31 samples were conducted at Majorbio Biotechnology Co., Ltd. (Shanghai, China), using the HiSeq 2500 platform (Illumina). Detailed descriptions of metagenomic sequencing, assembly, and binning are given in Supplementary Methods.

### Metagenomic community profiling

Alpha diversity analysis of microbial communities was performed using vegan package v2.5. Operational taxonomic units (OTUs) were extracted from the metagenomic data using SingleM v0.12.1 (https://github.com/wwood/singlem) by aligning to a database of 14 single-copy ribosomal proteins [35]. OTU tables were then summarized by rarefying and clustering using SingleM summarize. The Shannon index and Chao1 were calculated from the SingleM OTU tables across each of the single-copy marker genes using vegan package v2.5. To explore the microbial composition of each sample, 16S rRNA gene fragments were recovered from metagenomic raw reads using the phyloFlash v3.4 pipeline (parameters: -almosteverything) and classified with the SILVA v138 database [36]. For the beta diversity analysis using *rplB* OTU table, Bray–Curtis dissimilarity was calculated and visualized using a non-metric multidimensional scaling ordination (NMDS) plot [37].

### Functional annotation and phylogenetic analysis

For all contigs assembled from 31 metagenomic samples, gene calling was performed using Prodigal v2.6.3 (-p meta) and functional annotation was undertaken with METABOLIC v4.0 [38]. All predicted coding sequences were pooled and clustered at 95% nucleotide sequence similarity using CD-HIT v4.8.1 [39]. Finally, a total of 48,606,854 non-redundant gene clusters were obtained as the reference gene catalog. Taxonomic annotation was performed with the easy-taxonomy workflow in mmseqs2 (v13.45111) against the Genome Taxonomy Database (GTDB) [40]. For individual MAGs, only high-quality MAGs (completeness > 80%, redundancy < 10%) were selected for further analysis. The completeness of various metabolic pathways was determined using KEGGDecoder [41]. Key genes involved in carbon, nitrogen, sulfur, and other cycling were further identified using METABOLIC v4.0. The dbCAN web server was used for carbohydrate-active gene identification, which integrated three tools (HMMER, DIAMOND, and Hotpep) [42]. For phylogeny inference, protein sequences of functional genes were aligned using MAFFT (v7.490, -auto option) [43], and gap sequences were trimmed using trimAl (v1.2.59, -gappyout option). Maximum likelihood phylogenetic trees were constructed using IQ-TREE (v2.2.0.3) and visualized in Interactive Tree of Life (iTOL, v6) [44, 45].

### Abundance profiles

At the contig level, the relative abundances of genes related to carbon fixation and other metabolisms across 31 metagenomic samples were calculated from the non-redundant gene catalog using Salmon (v1.9.0) in the mapping-based mode (parameters: -validate Mappings -meta) [46]. Genes per million (GPM) values were used as a proxy for gene abundance, which were normalized based on the gene length and sequencing depth [37]. At the genome level, the relative abundance of each MAG was obtained using CoverM v0.6.1 in genome mode (parameters: -m relative_abundance –trim-min 0.10 –trim-max 0.90 –min-read-percent-identity 0.95 –min-read-aligned-percent 0.75 –min-covered-fraction 0) [37].

### Metatranscriptomic analysis

Total RNA was extracted from replicate samples of the metagenome analysis using the RNeasy PowerSoil Total RNA Kit (12,866–25, QIAGEN, Germany) according to the manufacturer’s instructions. Raw metatranscriptomic reads were quality filtered in the same manner as metagenomes. The reads corresponding to ribosomal RNAs were removed using SortMeRNA v.4.3.4 [47] with default parameters with the smr_v4.3_default_db database. Then, these high-quality metatranscriptomic reads were mapped to predicted protein-coding genes from the reference gene catalog and carbon-fixing MAGs using Salmon v.1.9.0 [46] in mapping-based mode (parameters: -validate Mappings -meta). The expression level of each gene was normalized to transcripts per million (TPM). Detailed protocols and analysis methods are given in Supplementary Methods.

### Statistical analyses

All statistical analyses were carried out in R v4.2.3. One-way ANOVA and Tukey’s test were conducted to analyze of spatial variations in DCF rates and environmental parameters, with significance set at *P* < 0.05. Pearson’s correlation analysis was performed to evaluate the relationships between environmental parameters and the DCF or depth using the R package linkET [48]. The redundancy analysis (RDA) was used to evaluate the linkage between environmental parameters and the abundance of different carbon-fixing pathways. All the analyses associated with microbial communities were performed using the R package vegan [49].

## Results and discussion

### Depth profile of DCF rates and physicochemical characteristics of mangrove sediments

**Fig. 1:**
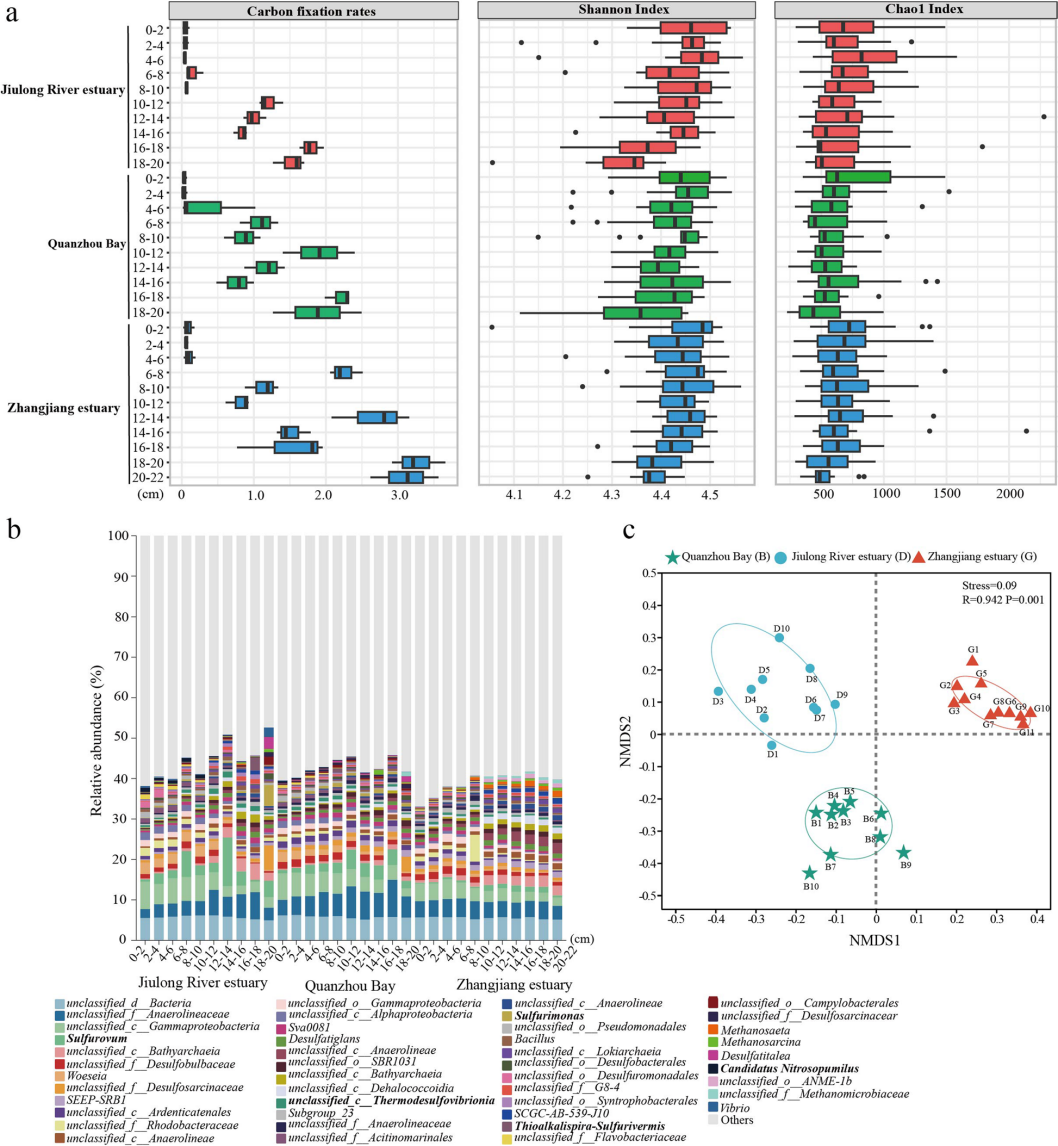
Dark carbon fixation (DCF) rates and microbial community compositions along the depth of different mangrove sediment sites. **a** The in-depth profile of DCF rates, Shannon index, and Chao1 index of microbial communities at three sample sites. Boxplots depict the 25–75% quantile range of the selected measurements, with the centerline depicting the median (50% quantile). Whiskers show the minimum and maximum values. **b** Relative abundance of genus-level bacterial and archaeal taxa based on 16S miTags extracted from the metagenomes. Only the top 45 genera, which account for more than 1% of the communities in at least one sample, are presented. The bold fonts represent the chemolithoautotrophic taxa. **c** NMDS analysis of a Bray–Curtis dissimilarity matrix calculated from the single-copy marker gene *rplB* OTU table. The difference significance of the community structures in the different samples was tested using ANOSIM with 999 permutations

Activity measurements by the ^14^C-labeled bicarbonate method were conducted to evaluate the DCF rates of 0–20 cm sediment layers from different mangrove sites in Fujian Province, China. Observed DCF rates fluctuated in the range of 0.02–3.21 mmol C m^−2^ day^−1^, and the average DCF rates were 1.09 mmol C m^−2^ day^−1^ across all 31 samples (Fig. 1a). DCF rates at the site of Zhangjiang estuary (0.07 to 3.21 mmol C m^−2^ day^−1^) were higher than those at the sites of Jiulong River estuary (0.04 to 1.77 mmol C m^−2^ day^−1^) and Quanzhou Bay (0.02 to 2.30 mmol C m^−2^ day^−1^) (Fig. 1a). These high DCF rates were identical to those reported in intertidal (0.27–3.37 mmol C m^−2^ day^−1^) and marine sediments (0.2–3.1 mmol C m^−2^ day^−1^) [24, 50], but higher than those of subtidal shelf (0.03–0.42 mmol C m^−2^ day^−1^), sub-Arctic sediments (0.08–0.6 mmol C m^−2^ day^−1^), and most lake waters or other freshwater [10, 51], and lower than those found in cascade reservoirs (1.5–14.7 mmol C m^−2^ day^−1^) [52]. Extrapolation of the DCF rates to the entire Fujian Province mangrove area, which is around 12.24 km^2^ [53], approximately 58.38 t of carbon is fixed by chemoautotrophs annually, indicating that the DCF process is crucial to the carbon cycle and cannot be overlooked. However, it should be noted that this estimate is merely possible, as mangrove areas can differ greatly across various geographic regions. Furthermore, the DCF rates showed a depth-dependent variability and increased along the depth, which reached a maximum at the 16–20 cm layers at all three sites (Fig. 1a). Additionally, the linear regression analysis indicated a significantly positive correlation between DCF rates and sediment depths (*R*^2^ = 0.807, *P* < 0.01, Fig. S3a).

We further investigate which environmental characteristics affect the DCF rates in mangrove sediments, examining their depth profiles at each site (Fig. S2). TC and TOC concentrations were high in all samples (0.92–2.13% and 0.69–1.01%, respectively), and decreased with the depth (Fig. S2 a, b). Low concentrations of TN (0.07–0.19%) and inorganic nitrogen, including NH_4_^+^, NO_3_^−^, and NO_2_^−^ (1.30–6.54 mg/kg, 0.14–0.95 mg/kg, and 0.01–0.09 mg/kg, respectively), were detected, which decreased consistently with the depth (Fig. S2 d–g). Similar ammonium-N concentrations (5.92–7.61 mg/kg) were detected in other mangrove sediment habitats, and the growth of mangrove trees was limited at such low ammonium-N concentrations [18, 54]. High concentrations of TS and AVS increased with depth, reaching a peak at 16–20 cm (1.42% and 2.86 mmol/L, respectively), whereas SO_4_^2−^ concentrations showed a reverse trend (Fig. S2 h–j). These results indicate that nitrogen is limited in these carbon- and sulfur-rich mangrove sediments, consistent with previous reports [18, 20]. The redox potential (Eh) of the top 0–4 or 0–6 cm layers at three sites was > 0 mV, indicating that the surface sediments were aerobic, and this value decreased sharply with depth, reaching the lowest value at the deeper layer (Fig. S2n). The highly reduced conditions in deeper sediments except surface sediments indicated that the mangrove sediments were mostly water-logged without much periodical aeration, which is consistent with the microbiological data. Among all the measured physicochemical parameters, TC, NH_4_^+^, NO_3_^−^, NO_2_^−^, SO_4_^2−^, moisture, salinity, and redox values showed negative linear correlations with the depth (*P* < 0.05), whereas DIC, TS, AVS, and pH showed positive correlations (*P* < 0.05) (Fig. S3). Furthermore, Pearson’s correlation analysis was used to explore the relationship between environmental parameters and the DCF rates. The results indicated that DCF rates were significantly correlated with DIC, TS, SO_4_^2−^, AVS, NH_4_^+^, NO_3_^−^, NO_2_^−^, moisture, pH, salinity, and redox (*R*^2^ = 0.571–0.675,* P* < 0.05; Fig. S4). However, no significant correlation was observed between DCF rates and organic carbon content (Fig. S4b), in line with the previous study [51]. Our results suggested that the depth-dependent variability in DCF rates within mangrove sediments was closely related to the environmental factors such as DIC, TS, AVS, NH_4_^+^, NO_3_^−^, and NO_2_^−^.

### Phylogenetic diversity of microbial community inhabiting mangrove sediments

To estimate the depth profile of mangrove sediment microbiomes, we extracted genomic DNA from the 31 sediment samples and performed metagenomic sequencing. Alpha diversities of bacterial and archaeal communities for each sediment core were performed based on 14 single-copy marker genes [37]. Overall, the results showed that the surface sediment at all three sites harbored the most diverse and richest microbial communities, with the highest values of Shannon (4.48 ± 0.07) at the 0–2 cm of Zhangjiang estuary, Chao1 (776.38 ± 274.85) at the 4–6 cm of Jiulong River estuary, and the highest bacteria cell density (3.09 × 10^9^ ± 4.47 × 10^8^ 16S rRNA genes g^−1^) at the 4–6 cm of Jiulong River estuary (Fig. 1a; S5). In contrast, the deeper sediments harbored the lowest microbial diversity and richness, with the values of Shannon (3.24 ± 0.08) at the 18–20 cm of Jiulong River estuary, Chao1 (495.17 ± 194.31) at the 18–20 cm of Quanzhou bay, and the highest archaeal cell density (5.67 × 10^8^ ± 1.64 × 10^7^ 16S rRNA genes g^−1^) at the 16–18 cm of Zhangjiang estuary (Fig. 1a; S5). Other middle sediments were compositionally similar to each other among sites and harbored moderately to highly diverse and abundant communities.

To profile the overall microbial community structure within these sediments, we classified 16S rRNA gene fragments recruited from metagenome raw reads for each sediment core. The dominant community members from bacterial phyla were *Pseudomonadota* (mainly the classes *Gamma-* and *Alphaproteobacteria*, on average 51.03% of the whole community), followed by *Chloroflexota* (6.95–23.39%, mainly the class *Anaerolineae*) and *Desulfobacterota* (7.78–22.32%, *Desulfobacteria*) (Fig. S6 a, b). Members from the phylum *Crenarchaeota* (1.18–6.60%, mainly the class *Bathyarchaeia*) were the most dominant archaea, followed by *Halobacterota* (0.11–6.86%, mainly the class *Methanosarcinia*), *Thaumarchaeota* (0.06–3.72%, *Thermoplasmata*), and *Nanoarchaeota* (0.38–1.24%, *Nanoarchaeia*) (Fig. S6 a, b). At the genus level, some canonical chemolithoautotrophic taxa occurred with the absolute dominance in different sediment samples (relative abundance > 1%), such as the sulfur- or/and hydrogen-oxidizing bacteria *Sulfurovum* (0.11–3.20%) and *Sulfurimonas* (0.03–5.22%) of the class *Campylobacteria*, the sulfur-oxidizing bacteria *Thioalkalispira*-*Sulfurivermis* (0.05–3.17%) of the class *Gammaproteobacteria*, the unclassified sulfate-reducing bacteria *Thermodesulfovibrionia* (0.42–1.33%), and ammonia-oxidizing archaea *Candidatus* Nitrosopumilus (0.01–1.07%) (Fig. 1b), suggesting that chemolithoautotrophs may play an important role in element cycles in situ. Furthermore, taxonomic profiles produced by 16S rRNA gene amplicon sequencing were broadly similar to metagenomic profiling, but with differences in the relative abundances of certain groups (Fig. S7). Beta diversity analysis based on the *rplB* OTU table using Bray–Curtis dissimilarity showed that the taxonomic compositions of these microbial communities were strongly correlated with sampling sites, followed by the sediment depth (ANOSIM, *R*^2^ = 0.942, *P* = 0.001) (Fig. 1c).

### The distribution and activity of carbon-fixing genes across the mangrove sediment depths

**Fig. 2:**
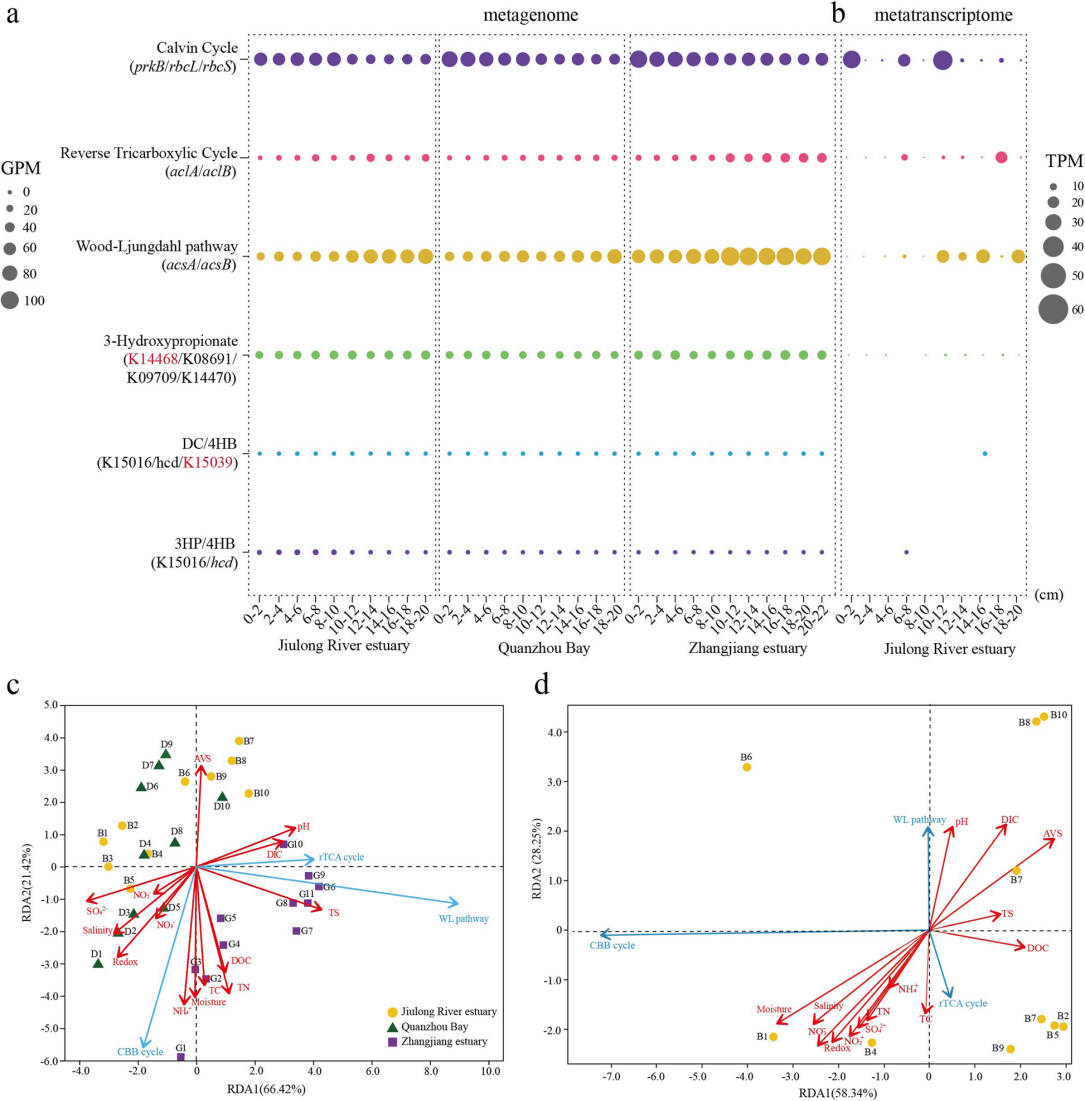
The abundance and expression of carbon fixation pathways along the depth at different mangrove sediment sites. **a** The average abundance of the carbon fixation marker genes at different depths of three sample sites by metagenomic analysis. **b** The average transcript expressions of the carbon fixation marker genes at different depths of three sample sites by metatranscriptomic analysis. *prkB*, phosphoribulokinase, K00855; *rbcL*, ribulose-bisphosphate carboxylase large chain, K01601; *rbcS*, ribulose-bisphosphate carboxylase small chain, K01602; *aclA*, ATP-citrate lyase alpha-subunit, K15230; *aclB*, ATP-citrate lyase beta-subunit, K152301; *acsA*, anaerobic carbon-monoxide dehydrogenase, K00198; *acsB*, acetyl-CoA synthase, K14138; 3-hydroxyacyl-CoA dehydrogenase, K15016; 4-hydroxybutyryl-CoA dehydratase, K14534; 3-hydroxypropionate dehydrogenase, K15039; malonyl-CoA reductase, K14468; malylCoA/(S)-citramalyl-CoA lyase, K08691; 3-methylfumaryl-CoA hydratase, K09709; 2-methylfumaryl-CoA isomerase, K14470. The orange marked genes mean the deletion in all samples. **c** RDA analysis of environmental parameters and the gene abundances of different carbon-fixing pathway. **d** RDA analysis of environmental parameters and the transcript abundances of different carbon-fixing pathway. Correlations between environmental variables and axes are represented by the length and angle of arrows. Colors and shapes represent the different habitats and depths

To understand the microbial metabolic strategy of inorganic carbon acquisition at different depths, we analyzed the distribution of marker genes (average abundance) representing different carbon fixation pathways in all samples. The related genes involved in six previously characterized carbon fixation pathways, including the CBB cycle, WL pathway, rTCA cycle, DC/4HB cycle, 3HP/4HB cycle, and 3HP bicycle, were detected. Among them, genes encoding the group I/II ribulose-1,5-bisphosphate carboxylase/oxygenase (RuBisCO) and phosphoribulokinase (*prk*), key enzymes in the CBB cycle, were ubiquitous throughout the sediment layers, but especially dominant (up to 95.34 GPM) in surface layers at all three sites (Fig. 2a). In contrast, the *acsA*/*acsB* genes, encoding carbon monoxide dehydrogenase/acetyl-CoA synthase associated with the WL pathway, were abundant and especially dominant (up to 104.05 GPM) at the deeper layers. Also, the rTCA cycle genes were strongly enriched in deeper sediment layers. The abundance of genes involved in the 3HP/4HB cycle was low (0.25–11.92 GPM) and decreased along the depth (Fig. 2a). In addition, the lack of partial marker genes of the DC/4HB and 3HP cycles indicated the incompleteness of these two pathways in mangrove sediments.

A quantitative assessment of all the key genes in the carbon-fixing pathways mentioned above suggested that potential CBB cycle (up to 59.48 TPM), followed by the WL pathway and rTCA cycle (up to 44.53 and 37.82 TPM, respectively), were the most active carbon fixation pathways within 0–20 cm layers of the mangrove sediments (Fig. 2b). Specifically, the transcripts encoding the CBB cycle were higher near sediment surface, whereas the transcripts encoding the WL pathway and rTCA cycle were more abundant in the deeper layers (Fig. 2b), which was consistent with the metagenomic results. Previous study has indicated that the WL pathway was more important in anaerobic conditions, challenging the conventional notion that the CBB cycle is the main DCF pathway in estuarine ecosystems [24]. These results suggest that spatial environmental gradients affect both the diversity and metabolic activity of carbon-fixing microbes in mangrove sediments.

Furthermore, the RDA analysis was conducted to understand the relationship between environmental factors and the average gene and transcript abundances of different carbon-fixing pathways (Fig. 2c, d). The results showed that the gene abundances of the CBB cycle were positively correlated to the NH_4_^+^, moisture, TC, TN, DOC, redox values, and salinity, which was largely consistent with the results of transcript abundances. The gene abundances of the rTCA cycle and the WL pathway were positively correlated to the pH, DIC, and TS. Furthermore, the transcript abundances of the rTCA cycle were positively correlated to the TC, and the WL pathway was positively correlated to the pH, DIC, and AVS (Fig. 2d). Previous research has demonstrated that environmental conditions can differently influence various microbial groups involved in carbon fixation. For example, in the Costa Rican convergent margin, the abundances of WL-containing MAGs were strongly correlated with higher iron and nickel concentrations, whereas that of CBB- and rTCA-containing ones were more associated with increasing DIC and phosphate concentrations [55]. In cascade reservoir sediments, *cbbL* and *cbbM* gene abundances were positively correlated to the TOC and inorganic elements including F^−^ and Cl^−^ [52]. Moreover, another study also indicated that temperature was associated with the *cbbM* gene abundance in coastal sediments [50]. Similarly, Zhao et al. (2020) documented that the average DCF rate in reservoir sediments was 36.6% higher at 25℃ than that at 15℃. These seasonal variations may be due to the effects of temperature on the metabolic activity of chemoautotrophs [52, 56].

### Potential carbon-fixing microorganisms identified within mangrove sediments

**Fig. 3:**
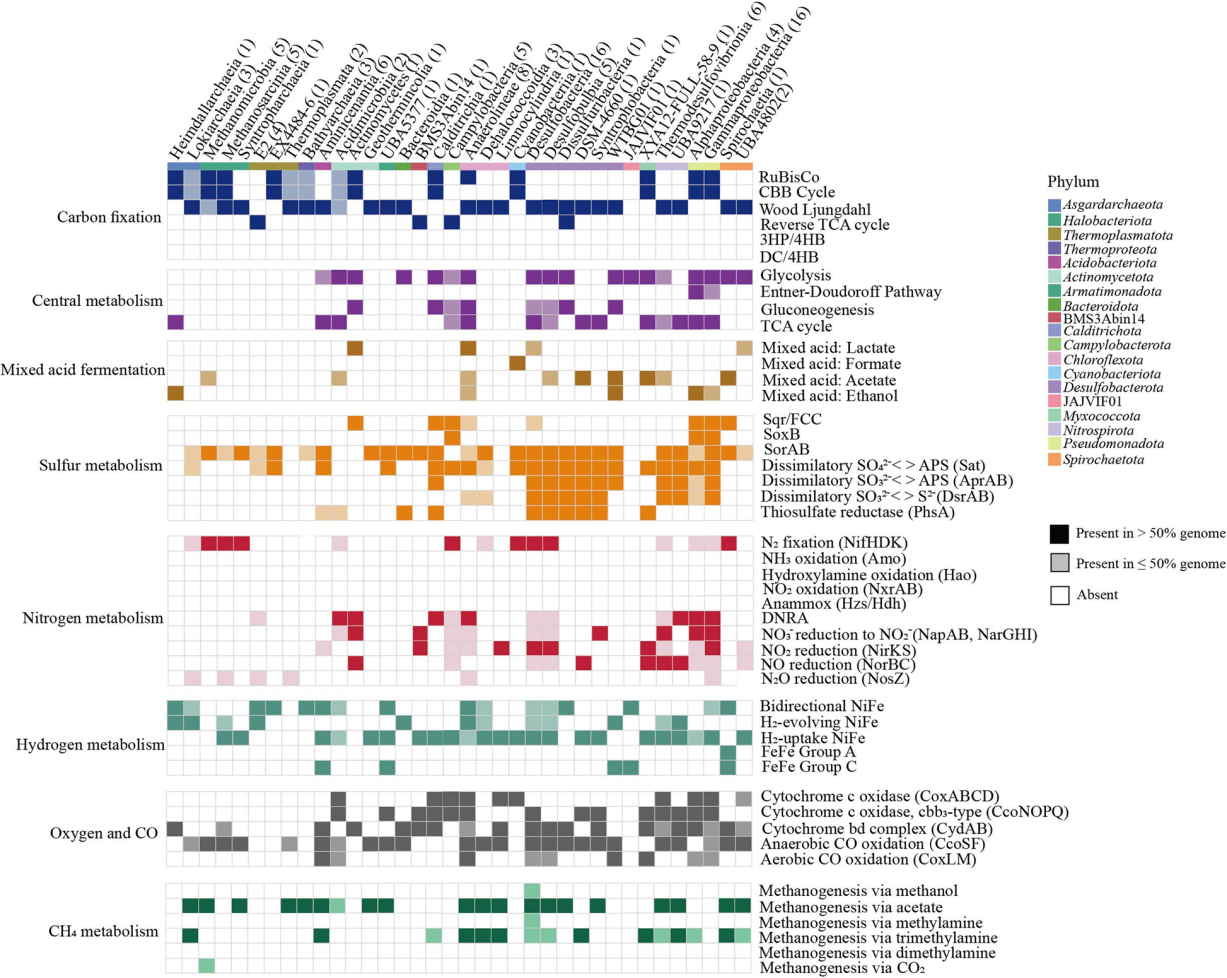
Pathways of carbon fixation identified in the 114 MAGs recovered from mangrove sediments. **a** Occurrence of core metabolic genes or pathways in phylogenetic clusters of autotrophic MAGs. Dark and light color shading indicate gene presence in > 50% and 1–50% of the genomes in each phylogenetic cluster, respectively. The number of MAGs per phylogenetic cluster is shown in brackets. Complete lists of metabolic genes or pathways can be found in Table S5 and S6

**Fig. 4:**
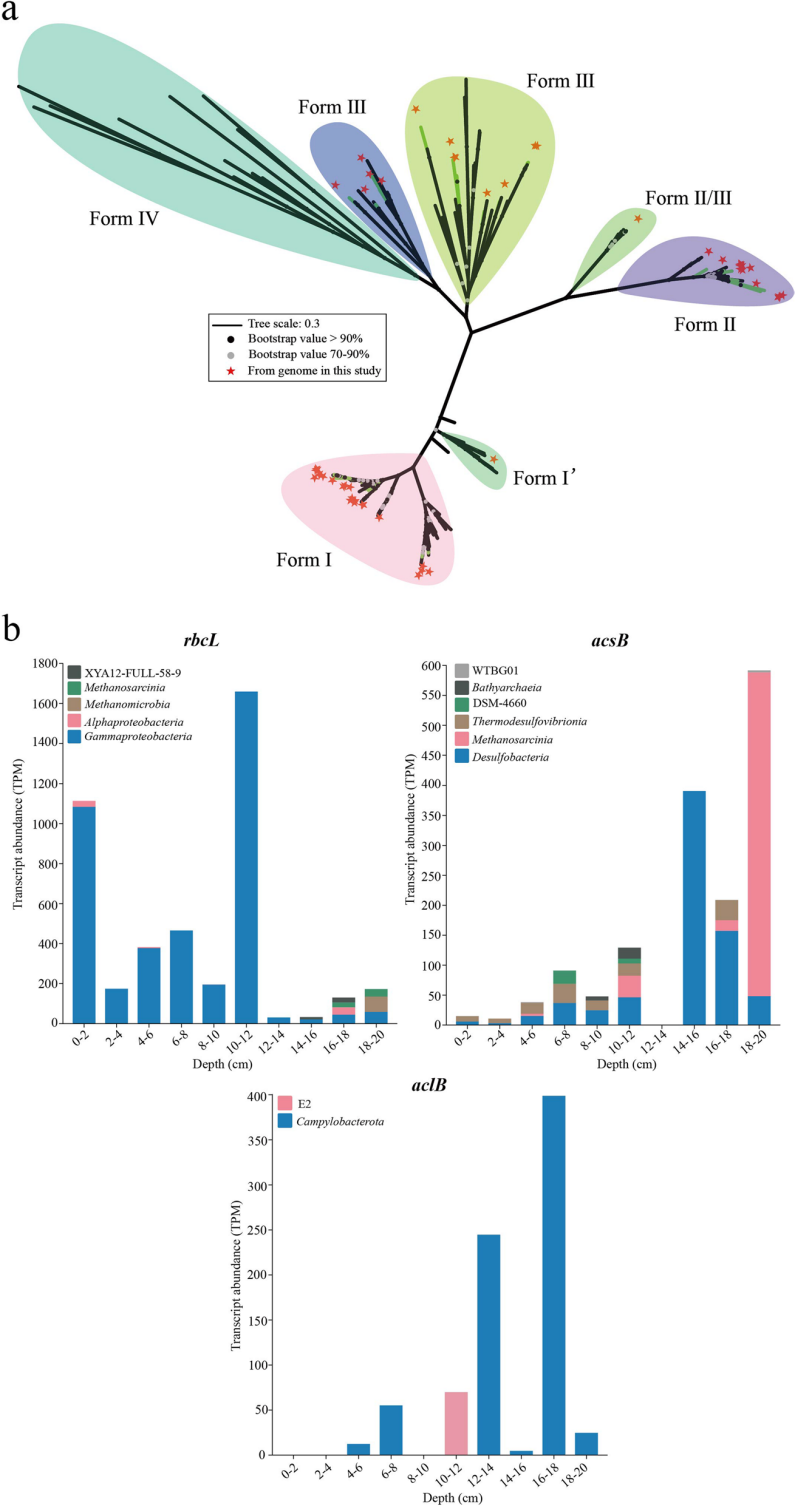
The phylogenetic analysis of key gene involved in the CBB cycle and the transcript expression of carbon-fixing pathway in mangrove sediments. **a** Maximum-likelihood phylogenetic tree of the RubisCO large-subunit RbcL detected in these carbon-fixing MAGs. For this trees, bootstrap values of > 70% are indicated as black circles at the nodes, and scale bars indicate the mean number of substitutions per site. **b** Transcription of genes involved in carbon fixation in all carbon-fixing MAGs at different sediment depths. The expression levels of each gene are represented in units of transcripts per million (TPM)

Through metagenomic assembly and binning strategies, we recovered 635 bacterial (*n* = 555) and archaeal (*n* = 80) genomes with > 50% completeness and < 10% contamination, which belonged to 48 phyla based on the GTDB taxonomy (Fig. S8; Table S1). Among these, a total of 114 high-quality MAGs (completeness > 80%, contamination < 10%) possessed the complete (completeness = 1) or partial (0.67 < completeness < 1) carbon fixation pathways, including the WL (*n* = 73), CBB (*n* = 39), and rTCA (*n* = 13) (Fig. 3; Table S2). Unexpectedly, the DC/4HB, 3HP/4HB, and 3HP cycles were incomplete in all carbon-fixing MAGs in this study. This indicated that these pathways could be more diversified than currently thought, or that an unknown pathway using shared enzymes with these cycles could be operating in the ocean. CBB cycle was found in 15 classes across 9 phyla (mainly *Pseudomonadota*, *Halobacteriota*, *Asgardarchaea*, *Actinomycetota*, and *Thermoplasmatota*) (Fig. 3). A phylogenetic tree of the RubisCO large subunits (RbcL) was constructed to determine the RubisCO forms present in this study, and the result indicated that most MAGs possessing the CBB cycle encoded form I or/and II RubisCOs (Fig. 4a). We also found a wealth of anaerobic or microaerophilic autotrophs that use energy-efficient but oxygen-sensitive carbon fixation pathways, such as the WL pathway and rTCA cycle. Genes encoding the WL pathway were found in MAGs encompassing 24 classes across 11 different phyla (mainly *Desulfobacterota*, *Chlorofexota*, *Acidobacteriota*, *Nitrospirota*, *Halobacteriota*, *Spirochaetota*, and *Thermoproteota*) (Fig. 3). Genes for the rTCA cycle occurred in MAGs from 4 classes across 4 phyla (*Campylobacterota*, *Thermoplasmatota*, BMS3Abin14, and JAJVIF01). Interestingly, several archaeal MAGs from *Asgardarchaeota*, *Halobacteriota*, *Thermoplasmatota*, and *Thermoproteota* possessed both the CBB cycle and WL pathways (Fig. 3). Previous study has indicated that these two carbon fixation pathways co-occurred in *Asgardarchaeota* and *Halobacteriota* from other mangrove sediment ecosystems [14]. Notably, our results represented the first genomic evidence of carbon-fixing potential in two bacterial candidate phyla JAJVIF01 and BMS3Abin14 (Fig. 3), which expanded the known diversity of carbon fixation taxa. Collectively, the results reveal diverse carbon-fixing prokaryotes in mangrove sediments, including two previously unreported phyla.

Based on reads mapping, the distributions of 114 MAGs of carbon-fixing microbes were further investigated by comparing to metagenomes obtained from different mangrove sediment samples (Fig. S9). The results indicated that each carbon-fixing microbe was usually not highly abundant, often comprising less than 0.5% of the microbial community across all samples (Table S3). When considered individually, chemoautotrophs using the CBB cycle were mainly affiliated with the *Gamma-* and *Alphaproteobacteria*, with relatively higher abundances at the 4–10 cm layers (Fig. S9). Among the microbes containing the WL pathway, *Desulfobacteria*, *Anaerolineae*, *Syntrophobacteria*, and *Thermodesulfovibrionia* were most abundant, and the relative abundances reached a peak at the layer of 18–20 cm. Furthermore, the abundances of *Campylobacteria* and the unclassified E2 lineage that encode the rTCA cycle were high at the 6–14 cm layers (Fig. S9). The dominant microbial taxa for different carbon-fixing pathways were consistent with the findings from the analysis of non-redundant gene clusters (Fig. S10). Our data thus suggests that these carbon-fixing microbes are widespread and seemly occupy distinct niches within mangrove sediment ecosystems.

To infer if the potential carbon-fixing microbes can fix carbon in situ, ten metatranscriptomes sequenced from mangrove sediments of the Jiulong River estuary were mapped against the above carbon-fixing MAGs (Fig. 4b). The results showed that most transcripts of *rbcL* gene encoding the CBB cycle, with an average of 419 reads, were mainly mapped to those of sulfur-oxidizing *Gammaproteobacteria* (mainly the genus *Thiodiazotropha*, Table S4), confirming their activity for autotrophs in situ. These transcripts were mainly found in the 0–12 cm layers with 191.88–1659.04 transcripts per million (TPM). The *acsB* transcripts involved in the WL pathway were strongly expressed at the 14–16 and 18–20 cm layers, and mainly affiliated with the sulfate-reducing *Desulfobacteria* (up to 390.78 TPM) and methane-producing *Methanosarcinia* (up to 540.54 TPM) (Fig. 4b). Additionally, the taxon *Desulfobacteria* was also highly expressed at the 16–18 cm layer with 157.51 TPM. The *aclB* transcripts for the rTCA cycle were mainly mapped on the class *Campylobacteria* (mainly the genus *Sulfurovum*) at the 12–14 and 16–18 cm, with the values 244.67 and 398.78 TPM, respectively (Table S4). These results suggest that although the CBB cycle plays an important role in DCF, as reported in the oceans and coastal sediments [57, 58], microbes with the WL pathway and rTCA cycle play a major role in deeper sediment layers, where they may represent a vast carbon sink.

### Sulfur oxidation and sulfate reduction are important metabolisms for carbon fixation in mangrove sediments

**Fig. 5:**
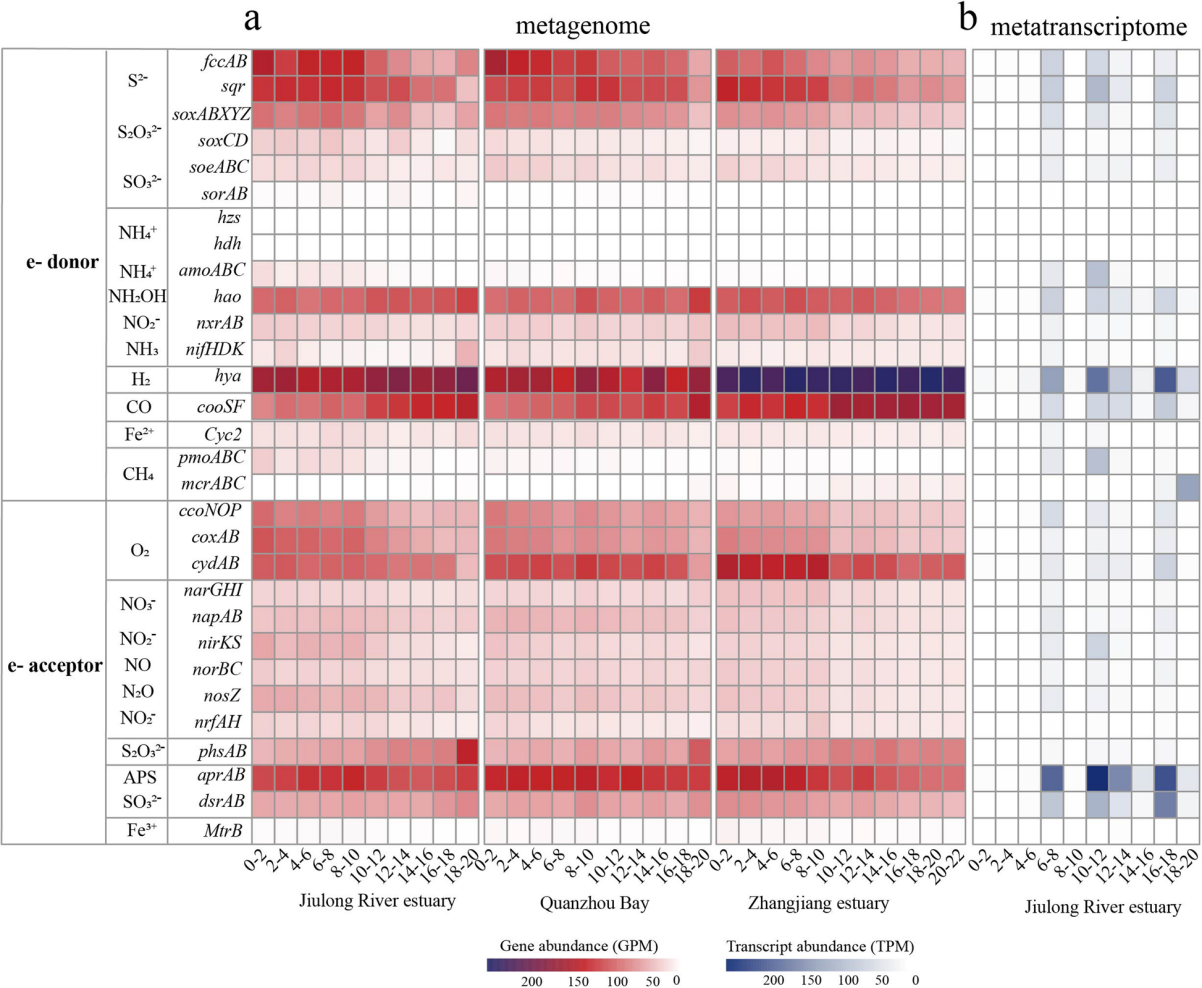
Abundance and expression of electron donors and acceptors that may couple with carbon fixation within the mangrove sediments. **a** The abundance of each makers gene for electron donors and acceptors at different depth in metagenomes. **b** The abundance of each transcript at different depth in metatranscriptomes. Gene and transcript abundances are represented in units of genes per million (GPM) and transcripts per million (TPM), respectively. Pathways that were not represented in a given sample remain white

**Fig. 6:**
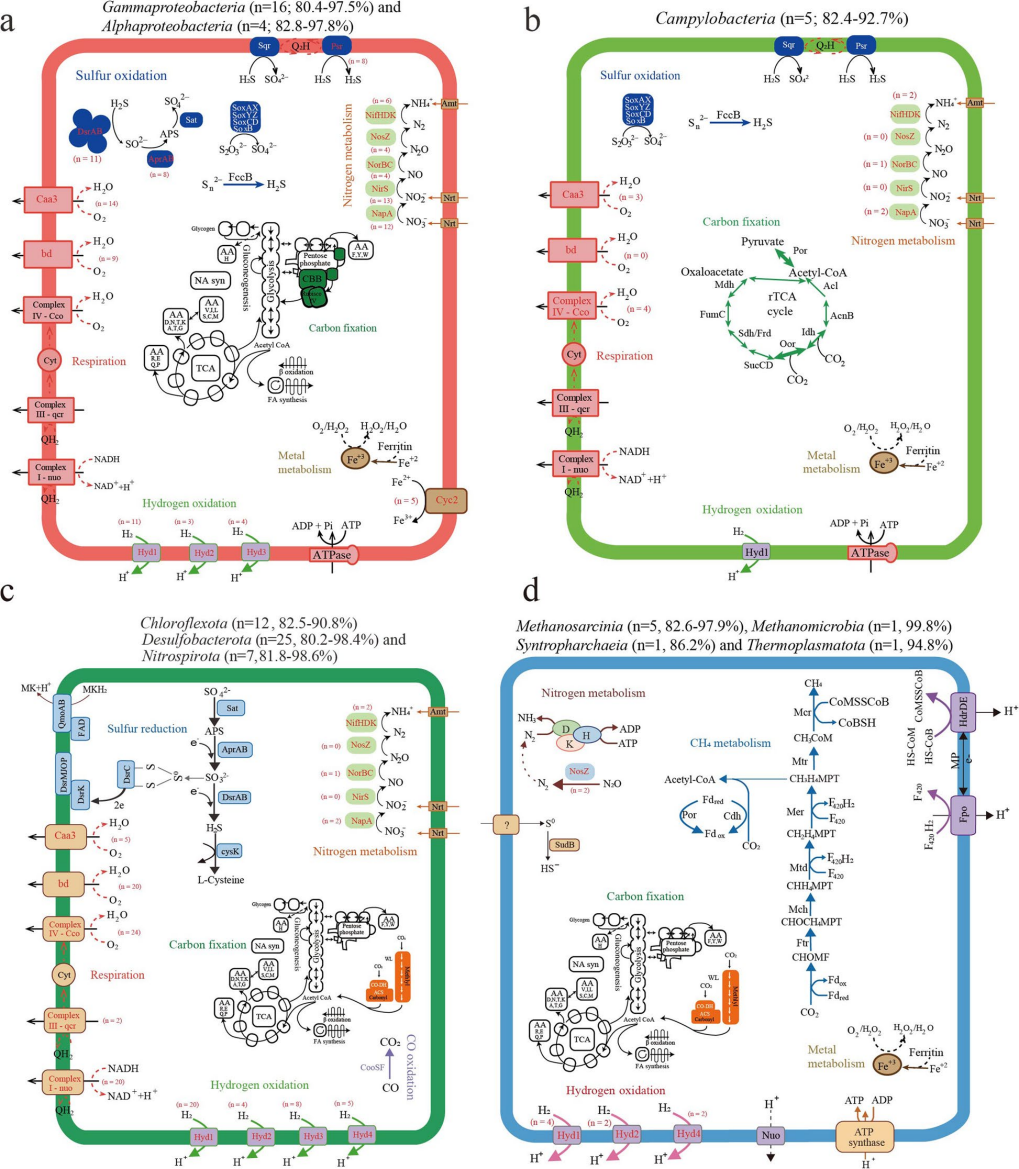
Metabolic reconstruction of core pathways for putatively representative carbon-fixing MAGs. **a** The sulfur-oxidizing *Gamma-* and *Alphaproteobacteria* with the CBB cycle. **b** The sulfur-oxidizing *Campylobacteria* with the rTCA cycle. **c** Dissimilatory sulfate reduction form *Chloroflexota*, *Desulfobacterota*, and *Nitrospirota* with the WL pathway. **d** Methanogenic archaea from *Methanosarcinia*, *Methanomicrobia*, *Syntropharchaeia*, and *Thermoplasmatota* with the WL pathway. Red font indicates that not all MAGs retrieved include the gene (numbers of MAGs with the corresponding gene indicated in parentheses). Comprehensive enzyme annotations are meticulously provided in Table S5 and S6

**Fig. 7:**
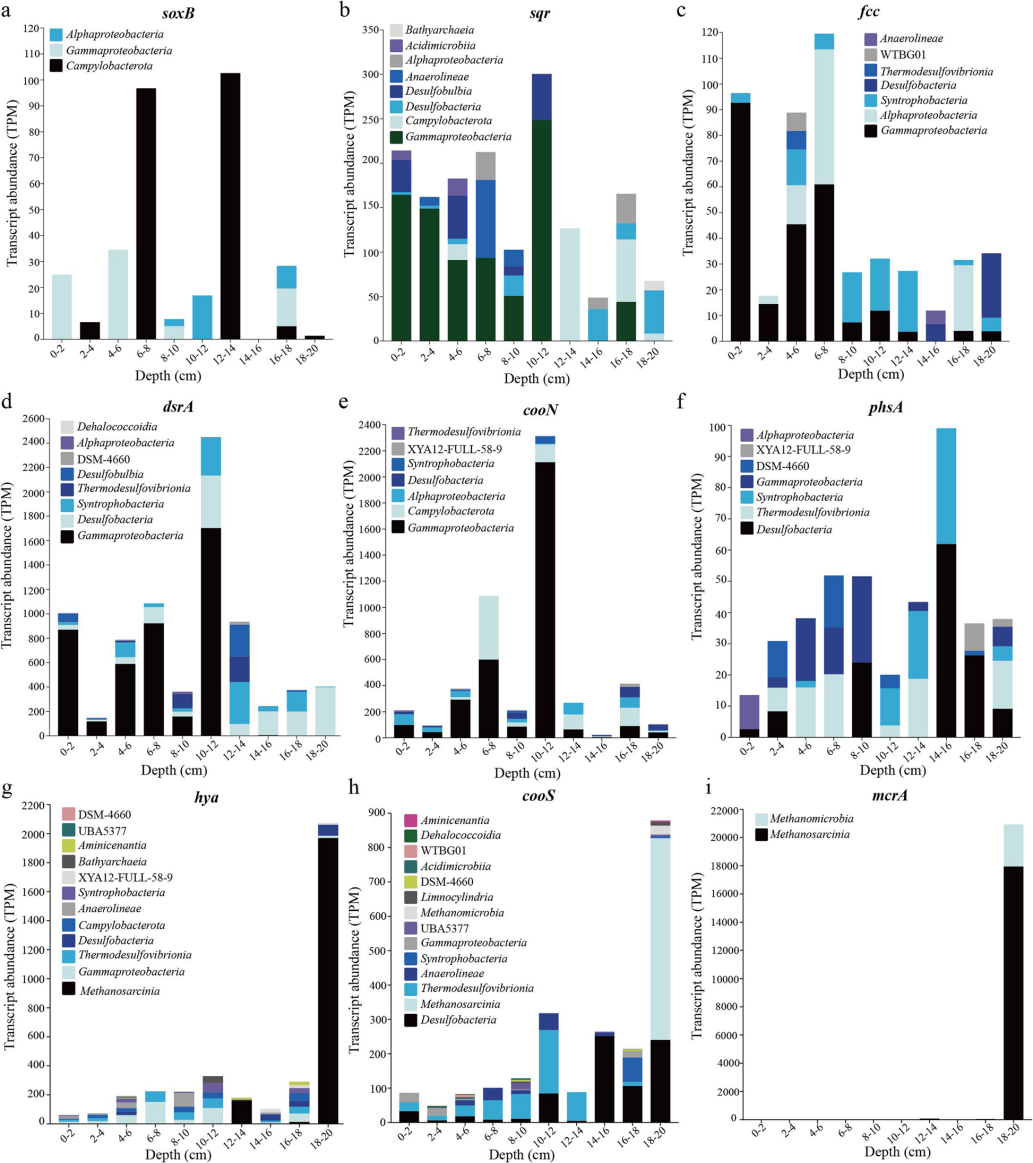
Transcription of genes of core metabolisms in all carbon-fixing MAGs at different sediment depths. Metabolic pathways included sulfur, nitrogen, oxygen, hydrogen, iron, methane, and other metabolisms. The expression levels of each gene are represented in units of transcripts per million (TPM)

Metabolically versatile autotrophs could couple successive redox to acquire energy for carbon fixation. Biogeochemical evidence has demonstrated that sulfur oxidation dominated chemoautotrophy in reducing substrates of coastal sediments [59]. In this study, metagenomic analysis indicated that genes responsible for sulfur oxidation (e.g., *fccAB* and *sqr* for sulfide oxidation, *soxABCDXYZ* for thiosulfate oxidation) were abundant and significantly (*P* < 0.05) decreased along the depth (Fig. 5a), which was consistent with the previous study [15]. Taxonomy classification based on function genes showed that these sulfur-oxidizing genes were mainly affiliated with the taxa *Gammaproteobacteria*, *Anaerolineae*, *Alphaproteobacteria*, *Campylobacteria*, and *Desulfobacteria* (Fig. S11 a–c). Furthermore, among the obtained 114 carbon-fixing MAGs, the groups with genomic potential for sulfur oxidation were also predominantly associated with *Gammaproteobacteria* (*Burkholderiales*, *Thiodiazotropha*, and *Thiomicrorhabdus*), *Alphaproteobacteria* (mainly the genera *Rhizobiales* and *Rhodobacterales*), and *Campylobacteria* (mainly the genus *Sulfurovum*) (Fig. 6 a, b; Table S5). The metabolic reconstruction showed that most of these MAGs contained the diverse oxygen reductases including *aa*_*3*_ and *cbb*_*3*_-type cytochrome c oxidases (*coxABCD* and *ccoNOQP*, respectively) and cytochrome *bd* ubiquinol oxidase (*cydAB*), indicating that these sulfur oxidizers likely utilize oxygen as a terminal electron acceptor. Among these MAGs, only two members of *Gammaproteobacteria* (b9_bin20 and g1_bin45) encoded a complete denitrification pathway (Table S5), which may be important contributors to both sulfur oxidation and denitrification in mangrove sediments. To identify the expression of sulfur oxidation pathways from the respective carbon-fixing microbes in situ, we mapped the metatranscriptomic reads to MAGs encoding *soxB*, *sqr*, *fcc*, and *dsrA* (Fig. 7 a–d). The results revealed that the major fraction (49–57%) of transcripts mapped to *sqr*, *fcc*, and *dsrA* sequences were affiliated with the class *Gammaproteobacteria*, and expressed mainly at the 0–10-cm layers, further supporting their central roles in chemolithoautotrophy at surface sediments. The transcript of *soxB* from *Gammaproteobacteria* was only expressed at the layers of 0–2, 4–6, and 8–10 cm with low values of 5.07–34.52 TPM (Fig. 7a), indicating that thiosulfate oxidation plays a minor role for this taxon. The reverse dissimilatory sulfate reduction (rDSR), whose energy yield is higher than that of the Sox complex [60], was most well expressed by *gammaproteobacterial* SOB (up to 1703.2 TPM, largely exceeding expression of *soxB*, *sqr*, and *fcc* genes). Thus, these data suggest that reverse dissimilatory sulfate reduction is more active than sulfur oxidation for *Gammaproteobacteria* in DCF at these sites. Furthermore, the transcript of *soxB* was mainly affiliated with *Campylobacteria* (up to 102.63 TPM) at 12–14 and 16–18 cm layers, together with *sqr* transcript (up to 126.67 TPM), suggesting that thiosulfate and sulfide may be important energy sources for this taxon in situ. Among three oxygen reductases in these sulfur-oxidizing MAGs, a high number of *ccoN* transcripts were identified, an enzyme that supports microaerobic respiration, and possibly play a role in oxygen scavenging to prevent poisoning [61]. These transcripts were mainly expressed in *Gammaproteobacteria* (up to 2112.41 TPM) at 0–12-cm layers, *Campylobacteria* (up to 485.92 TPM) at 12–14- and 16–18-cm layers, and *Alphaproteobacteria* (up to 88.94 TPM) at 0–2-cm layers (Fig. 7e)*.* This is beneficial to those microorganisms, as mangrove sediments often fluctuate between oxic and anoxic conditions over short distances and timescales due to pore-water advection and physical disruptors as induced by waves and currents [7, 18]. Moreover, the transcripts of *napA*, *norB*, *nirS*, and *nosZ* involved in denitrification were much lowly expressed in all sediment layers and the corresponding carbon-fixing MAGs (Fig. 5b; S12 c–f), indicating that this pathway may be insignificant for these SOB in mangrove sediments.

Anaerobic sulfate reduction is considered as the most important respiration process in marine and mangrove sediments, participating in the mineralization of organic matter and releasing reducing inorganic compounds [8]. In this study, the relative abundance of *aprAB* for dissimilatory sulfate reduction significantly (*P* < 0.05) increased along the depth (77.26–135.32 GPM), which was consistent with the previous study [15]. However, the relative abundance of *dsrAB* did not show significant differences along the sediment depth (Fig. 5a), probably because both genes are also involved in multi-step processes of sulfur oxidation as a reverse reaction for sulfate reduction [62]. The major taxa containing *dsrAB* were found in the carbon-fixing MAGs from sulfur-oxidizing bacteria (SOB) (dominated by *Burkholderiales*, *Thiodiazotropha*, and other *Gammaproteobacteria*), and sulfate-reducing bacteria (SRB) (dominated by *Desulfobacteria*, *Desulfobulbia*, and *Thermodesulfovibrionia*) (Fig. 6a, c), which was similar to the main microbial taxa from the result of non-redundant gene clusters (Fig. S11d). Metatranscriptomic analysis indicated that the *dsrAB* transcripts were highly expressed at deeper layers (Fig. 5b). Furthermore, based on the transcript analysis of gene *dsrA* in all carbon-fixing MAGs, most of them were assigned to the taxa from *Desulfobacteria* (up to 430.54 TPM) at 14–18 cm, *Syntrophobacteria* (up to 344.31 TPM) at 10–12 cm, *Thermodesulfovibrionia* (up to 208.47 TPM) at 12–14 cm, and *Desulfobulbia* (up to 261.31 TPM) at 12–14 cm layers, respectively (Fig. 7d). Thus, our study highlights that these SRB may play important ecological roles for carbon fixation in the deeper sediment layers. Genome analysis indicated that they also contained the genes encoding oxygen reductase such as *cco* and *cyd*, and the partial denitrification pathway such as *napA*, *norBC*, *nirKS*, or *nosZ* (Fig. 6c); however, these gene transcripts were not expressed or at very low levels, suggesting that oxygen and nitrate are not the main electron acceptors for these SRB in situ. Furthermore, we found the transcripts of *phsA* responsible for sulfur disproportionation mainly assigned to the MAGs from the classes *Desulfobacteria* (up to 61.92 TPM) and *Syntrophobacteria* (up to 51.74 TPM) (Fig. 7f). This indicated that sulfur disproportionation might be an important energy source for sulfate-reducing *Desulfobacterota*, which was consistent with the previous study [63].

### Hydrogen and CO are the overlooked energy sources for carbon fixation in mangrove sediments

Previous study indicated that genes coding for uptake [NiFe]-hydrogenase were found in metagenomic bins of sulfur-oxidizing *Gammaproteobacteria* from estuarine sediments, indicating alternative energy sources for dark carbon fixation under oxic to suboxic conditions [64]. Here, we tested whether hydrogen could serve as an alternative energy source also for sulfur oxidizers in mangrove sediments. Metagenomic analysis demonstrated a significant presence of genes encoding hydrogenases (*hya*), which were especially dominant (up to 246.89 GPM) at the deeper layers (Fig. 5a). The genes encoding diverse groups 1 to 4 NiFe hydrogenases were mainly found in the carbon-fixing MAGs of *Chloroflexota*, *Desulfobacterota*, *Pseudomonadota*, *Nitrospirota*, *Campylobacterota*, and *Thermoplasmatota* (Table S5; S6), which was consistent with the main microbial lineages from the non-redundant gene clusters (Fig. S11e). Several MAGs from *Chloroflexota*, *Nitrospirota*, *Desulfobacterota*, and *Halobacteriota* encoded both H_2_-uptake (group 1) and H_2_-producing (group 4) NiFe hydrogenases, suggesting the putative genomic capabilities of both H_2_ production and consumption of these taxa. Metatranscriptomic analysis indicated that in addition to the highest transcripts of hydrogenase from hydrogenotrophic *Methanosarcinia* occurring at the 18–20 cm (up to 1970.18 TPM), a substantial fraction (29%) of all hydrogenase transcripts were assigned to the sulfur-oxidizing bacteria *Gammaproteobacteria* (up to 151.69 TPM) at 6–12 cm and *Campylobacteria* (up to 53.63 TPM) at 16–18 cm (Fig. 7g). Although the expression levels of hydrogenases in these sulfur-oxidizing taxa were lower than those of sulfur-oxidizing genes, they were in the same order of magnitude. Thus, hydrogen is also the important energy source for carbon fixation in sulfur-oxidizing *Gammaproteobacteria* and *Campylobacteria* in mangrove sediments.

Besides hydrogen, CO is another ubiquitous atmospheric trace gas produced by natural and anthropogenic sources, and has been reported as an important carbon and energy source in various habitats such as hot springs, soils, and hydrothermal vents [65, 66]. In this study, the abundances of anaerobic CO dehydrogenase (*cooSF*, K00196, and K00198) were high, especially dominant (up to 153.08 GPM) at the deeper layers (Fig. 5a). The *cooS* gene was mostly affiliated with the taxa *Desulfobacterota*, *Chloroflexota*, *Planctomycetota*, *Nitrospirota*, and *Actinomycetota* (Fig. S11f). Anaerobic conversion of CO has been reported for carbon assimilation pathways in sulfate-reducing bacteria [66]. The co-occurrence of CO dehydrogenase and sulfate reductase was observed in carbon-fixing MAGs of *Desulfobacterota*, *Chloroflexota*, and *Nitrospirota* (Fig. 6c), suggesting that they may potentially couple CO oxidation with sulfate reduction to derive energy for carbon assimilation through the WL pathway. Metatranscriptomic analysis indicated that the transcripts of *cooSF* were also highly expressed at the deeper layers (Fig. 5b). Moreover, *CooS* transcript for anaerobic CO oxidation was mainly affiliated with *Desulfobacteria* (up to 252.12 TPM) at 14–20 cm, *Methanosarcinia* (up to 586.84 TPM) at 18–20 cm, and *Thermodesulfovibrionia* (up to 184.77 TPM) at 10–12 cm, respectively (Fig. 7h). These results suggested that anoxic CO oxidation might also be an important energy supplement for carbon fixation in mangrove sediments, which is produced from the degradation of organic matter in the deeper layers by heterotrophs [18].

### The key roles of methanogenesis and other metabolisms for carbon fixation in mangrove sediments

Methanogens utilizing different substrates for methanogenesis have been reported to exist in various anaerobic marine and freshwater sediments [67]. Although SRB are known to outcompete methanogens over common substrates like acetate and H_2_, directing the electron flow towards CO_2_ rather than methane [68], several studies showed that SRB and methanogens could co-exist under high sulfate concentrations, especially in estuarine and mangrove sediments [69, 70]. In this study, the relative abundance of the key *mcrA* gene encoding methyl coenzyme M reductase related to methane production was low (0.06–11.55 GPM) but significantly (*P* < 0.05) increased along the depth (Fig. 5a), which was consistent with the previous study [15]. Both the taxonomic classification of gene *mcrA* and carbon-fixing MAGs showed that the main taxa responsible for methanogenesis were mainly affiliated with *Methanosarcinia*, *Methanomicrobia*, *Syntropharchaeia*, and *Thermoplasmatota* (Fig. 6d; S13). Metatranscriptomic analysis showed that the *mcrA* transcript for CH_4_ production was generally low, but exhibited the highest values at the 18–20 cm (Fig. 5b), suggesting a possibly more active methanogenesis in the deep layers, which was consistent with the previous study [71]. The high *mcrA* transcript was mainly mapped to *Methanosarcinia* and *Methanomicrobia* with the values of 17,954.64 and 2972.27 TPM, respectively (Fig. 7i), indicating that these taxa seem to be the primary methanogens driving carbon fixation in situ. Notably, substantial expressions of the iron reduction *mtrA* gene were mainly affiliated with *Methanosarcinia* (1442.17 TPM at 18–20 cm, Fig. S12g), supporting the link between iron reduction and methanogens, as shown previously in pure cultures such as *Methanosarcina barkeri*, *M. voltaei*, and *M. maze* [72, 73]. However, the relative abundance of particulate methane monooxygenase (*pmoA*), which is involved in methane oxidation, was low (0.68–26.44 GPM) and decreased along the depth (Fig. 5a), and the taxa responsible for methane oxidation were not found in any carbon-fixing MAG (Table S5), indicating that the aerobic methanotrophs may play a minor role in mangrove sediments of 0–20 cm.

Other energy-conserving metabolisms, such as anammox, nitrification, and iron oxidization, which may fuel carbon fixation in mangrove sediments, are also investigated. Previous studies have indicated that ammonia oxidation was suggested to play only a minor role in chemoautotrophy in coastal sediments [24, 59]. Metagenomic analysis revealed that the abundances of anammox genes (e.g., *hzs* and *hdh*) were too low to be detected in all samples, and could not be retrieved in any carbon-fixing MAG (Fig. 3; 5a), suggesting that the anammox process is insignificant in chemoautotrophic carbon fixation in mangrove sediments. Furthermore, nitrification genes, including *amo*, *hao*, and *nxrAB*, were detected with relatively high abundances (Fig. 5a), and they mainly affiliated to the taxa *Gammaproteobacteria*, *Nitrososphaeria*, *Nitrospiria*, *Anaerolineae*, and *Actinomycetota* (Fig. S11g; S14; S15). However, they failed to be found in any carbon-fixing MAG including the typical nitrite-oxidizing bacteria *Nitrospirota* [74, 75], possibly due to genome incompleteness. Additionally, metagenomic analysis indicated that a low abundance (8.82–19.94 GPM) of iron-oxidizing gene (cytochrome c, *cyc2*) was detected, and the taxa responsible for iron oxidation were rarely obtained from the metagenomes and carbon-fixing MAGs, appearing to be predominated by *Pseudomonadota*, *Acidobacteriota*, *Gemmatimonadetes*, and *Chloroflexota* (Fig. 5a; S11h; Table S5). Metatranscriptomic analysis indicated the gene *cyc2* for iron oxidation was much lowly expressed at all samples, and the transcript was only affiliated to *Gammaproteobacteria* (4.94 TPM), indicating its minor role in mangrove sediments (Fig. 5b; S12h). Overall, these results demonstrated that the metabolisms such as anammox, nitrification, and iron oxidization might play minor roles for carbon fixation in mangrove sediments.

### Mixotrophic lifestyle of carbon-fixing microbes in mangrove sediments

**Fig. 8:**
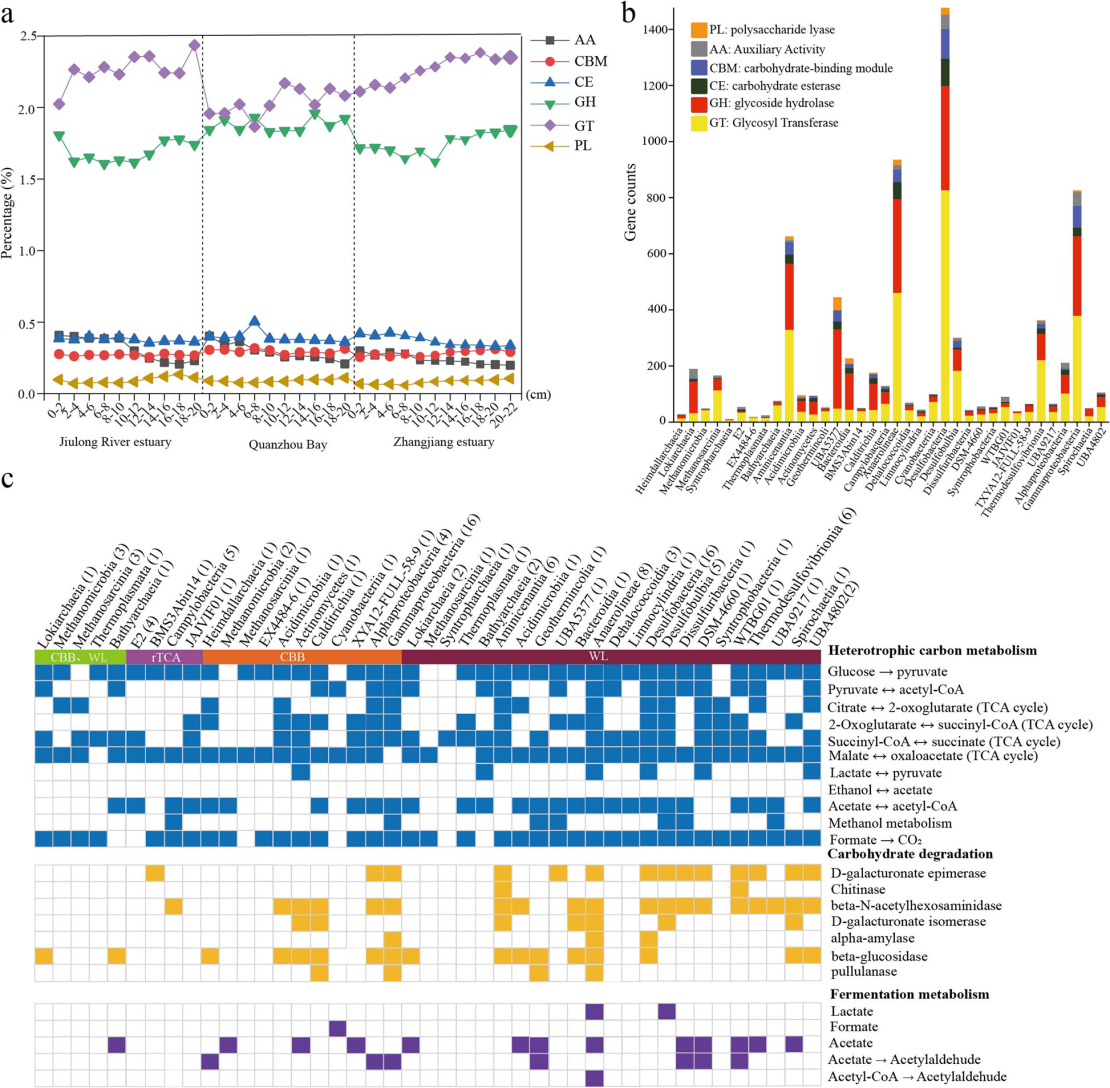
Utilization of carbohydrate-active enzymes (CAZymes) and substrate for all carbon-fixing microorganisms in mangrove sediments. **a** Distribution of CAZy genes along the depth in metagenome of all sediment samples, including auxiliary enzymes (AA), carbohydrate-binding modules (CBM), carbohydrate esterases (CE), glycoside hydrolases (GH), glycosyltransferases (GT), and polysaccharide lyases (PL). **b** The number of CAZy genes in all carbon-fixing MAGs. The different CAZy families represented include AA, CBM, CE, GH, GT, and PL. **c** Filled boxes indicate the presence of the metabolic process or bioenergetic complex in the corresponding genome while empty boxes indicate its absence. A metabolism was considered present only if the marker genes encoding full enzymatic pathway capable of carrying out that metabolic process were found in the genome

In addition to the capacity to fix inorganic carbon, some autotrophic microorganisms are probably able to utilize a variety of organic carbon compounds [76, 77]. Analysis of CAZymes in metagenomes and MAGs revealed that these microbes may have large potential for organic carbon degradation (Fig. 8). Glycoside hydrolases (GHs) and glycosyltransferases (GTs), which play key roles in the breakdown to polymeric substrates [78], were the most abundant at all sediment layers, and their relative abundances increased along the depth (Fig. 8a; Table S7, S8). Intriguingly, MAGs harboring the WL pathway affiliated to *Desulfobacteria*, *Anaerolineae*, and *Aminicenantia* encoded a broader repertoire of CAZymes, with the highest relative abundances of GHs and GTs (Fig. 8b; Table S9). It has long been believed that the WL pathway is used in diverse metabolic processes, and is best suited for mixotrophy [79, 80]. Microbes with the WL pathway in mangrove sediments mainly displayed a mixotrophic lifestyle, with a considerable diversity of energy metabolisms including central carbon metabolisms and fermentation (Fig. 8c; Table S10). The central carbon metabolism pathways included glycolysis, TCA pathway, and gluconeogenesis. The majority of MAGs harbored a complete TCA pathway and utilized oxidative phosphorylation to produce ATP with complex I (*nuo*) and complex II (*sdh*/*frd*) being widely distributed (Table S10). The *ldhA* gene for metabolic conversion of lactate and pyruvate was found in ten MAGs assigned to *Bathyarchaeia*, *Actinomycetes*, *Anaerolineae*, *Desulfobacteria*, *Syntrophobacteria*, and *Spirochaetota*. Only the MAGs from *Acidobacteriota*, *Armatimonadota*, and *Desulfobacterota* harbored the genes for formate formation from pyruvate (formate C-acetyltransferase, *pflD*), whereas the majority of MAGs contained the genetic potential to oxidize formate to CO_2_ by possessing the *fdh*/*fdo* gene (Table S10). The key genes of acetate metabolism were detected in ten MAGs mainly affiliated to *Methanomicrobia*, *Acidimicrobiia*, *Anaerolineae*, *Desulfobacterota*, and *Myxococcota* (Fig. 8c). Most of these acetogenic bacteria are known to be metabolically flexible utilizing various substrates for heterotrophy, including one-carbon compounds (formate, methanol), two-carbon compounds (glycolate, oxalate), lactate, and pyruvate [81]. Thus, these mixotrophs with the WL pathway could assimilate organic compounds in addition to the fixation of CO_2_, and had a competitive advantage over obligate autotrophs or heterotrophs in mangrove sediments. Notably, there are microorganisms with the presence of two different autotrophic pathways in this study, and they not only encoded genes for the WL pathway, but also contained genes required for CO_2_ fixation via the CBB cycle (Fig. 3; 4b). We thus speculate that the conditional usage of different CO_2_ fixation pathways may be particularly advantageous for those microbes living in dynamic mangrove sediment habitats. When in a high-energy situation, organisms would fix CO_2_ via the CBB cycle or use it as an alternative biosynthesis pathway of ribulose1,5-bisphosphate in archaea that is generally deficient in phosphate dikinase, while under low-energy conditions, they would switch to the energetically more favorable WL pathway, likely previously described in the world’s deepest blue hole [76]. More recently, two carbon fixation pathways of CBB and rTCA cycles were found in chemoautotrophic tubeworm *Riftia pachyptila*, and both of which were actively expressed, which conferred their advantage in the dynamic hydrothermal vents [82]. These results indicated that the microbial communities in deeper sediment layers appeared to be mixotrophic with a highly flexible carbon acquisition strategy.

## Conclusions

The findings from this research significantly enhance our understanding of dark carbon fixation within coastal eutrophic sediments, shedding light on the ecological significance of chemolithoautotrophic microorganisms in carbon cycling. High DCF rates in mangrove sediments indicate that the DCF process is crucial to the carbon cycle and cannot be overlooked. The most abundant chemolithoautotrophs in mangrove sediments are *Gammaproteobacteria*, *Desulfobacteria*, and *Campylobacteria*, accounting for about 52% of total chemoautotrophs. It is worth noting that although the CBB cycle plays an important role in DCF in mangrove sediments as described in coastal sediments, the WL pathway and rTCA cycle play more significant roles in deeper sediment layers. Chemolithotrophy appeared to be primarily driven by the oxidation of reduced sulfur species, hydrogen, and CO as well as the methanogenesis along the depth, with oxygen, sulfur, and iron as the terminal electron acceptor. Other energy-yielding processes, such as methane, nitrite, ammonia, or iron oxidation, were presumably contributing less to chemolithoautotrophy. Furthermore, microbes with the WL pathway always adopt a mixotrophic strategy for carbon assimilation. Overall, this study sheds light on the ecological importance and underlying mechanisms of the DCF process driven by chemoautotrophs in carbon-rich mangrove sediments, and further provides a vital basis for the understanding of microbial-mediated carbon cycle in the global oceans. However, it also should be noted that mangroves in different geographical regions may harbor distinct microbial communities and carbon fixation mechanisms. Future studies with the incorporation of biological replicates in meta-omics analysis and the exploration of deeper sediment layers (> 22 cm) across additional mangrove sites (e.g., Hainan, Guangdong, and Guangxi Provinces), as well as integrating other methods (e.g., stable isotope probing, microscopy, and FISH-nanoSIMS), are needed to better verify the contribution of DCF in this “blue carbon” ecosystem, further elucidating its global role in carbon sequestration.

## Supplementary Information

Supplementary Material 1.

Supplementary Material 2.

## Data Availability

All metagenomic and metatranscriptomic raw reads used in this study are available in NCBI under BioProject numbers PRJNA1017975 and PRJNA1170003 as well as PRJNA1018229, respectively. The present study did not generate codes, and the mentioned tools used for the data analysis were applied with default parameters unless specified otherwise.
