# Supplementary material for: Phylogenetically and metabolically diverse active carbon-fixing microbes reside in mangrove sediments
Source: Microbiome. 2025 Sep 1;13:194. doi: 10.1186/s40168-025-02177-9 (PMC12400630; doi:10.1186/s40168-025-02177-9)
Supplement: Supplementary file 2 — Supplementary Material 1. [file 40168_2025_2177_MOESM1_ESM.pdf]

Supplementary Information for

Phylogenetically and metabolically diverse active carbon-fixing microbes reside in mangrove sediments

Shasha Wang[1], Zhuoming Zhao[1], Ruolin Cheng[1], Liang Cui[1], Jun Wang[1], Maxim Rubin-Blum[2], Yao Zhang[3], Bolin Liu[4], Xing Chen[5], Federico Baltar[6], Xiaxing Cao[1], Xuezhe Wen[1], Karine Alain[7], Zhen Chen[1], Jing Liao[1], Lijing Jiang[1,8*] and Zongze Shao[1,8*]

[1] Key Laboratory of Marine Genetic Resources, Third Institute of Oceanography, Ministry of Natural Resources of PR China, Xiamen 361005, China

[2] Biology Department, National Institute of Oceanography, Israel Oceanographic and Limnological Research (IOLR), Haifa 3108000, Israel

[3] State Key Laboratory of Marine Environmental Sciences, College of Ocean and Earth Sciences, Xiamen University, Xiamen 361101, China

[4] State Key Laboratory of Estuarine and Coastal Research, East China Normal University, Shanghai 200241, China

[5] Frontiers Science Center for Deep Ocean Multispheres and Earth System, and College of Marine Life Sciences, Ocean University of China, Qingdao 266003, China

[6] Shanghai Engineering Research Center of Hadal Science and Technology, College of Marine Sciences, Shanghai Ocean University, Shanghai 201306, China

[7] Univ Brest, CNRS, Ifremer, EMR6002 BIOMEX, Biologie Interactions et adaptations des Organismes en Milieu EXtrême, IRP 1211 MicrobSea, Plouzané F-29280, France

[8] Fujian Ocean Innovation Center, Xiamen 361102, China

*Correspondence: Lijing Jiang (jianglijing@tio.org.cn)

Zongze Shao (shaozz@163.com)

Contents of this file

Supplementary Methods

Supplementary Figs S1 to S15

Supplemental Methods

Dark carbon fixation rates

DCF rates were measured through the assimilation of $NaH^{14}CO_3$ tracer (58.0 mCi $mmol^{-1}$, PerkinElmer, USA) [1, 2]. In brief, sediment slurries were made by mixing fresh sediment and sterile filtered (0.22 μm) overlying water at a ratio of 1:1 (wt/vol) in an anaerobic chamber. 1 ml of slurry along with a formaldehyde-killed control, was incubated in triplicate in darkness at the *in situ* temperature following the addition of 3 μCi of $NaH^{14}CO_3$ tracer. After 24 h, incorporation of DIC was terminated by adding 2% formaldehyde. The samples were then centrifuged at 12,000 g for 5 min, and the supernatant was discarded, and the remaining sediment pellets were washed for three times with 1 × phosphate-buffered saline containing 23 mM sodium bicarbonate. 1 ml of 3 M HCl was then added into the samples, which were transferred into a new 50 ml-centrifuge tube. 0.5 ml HCl was added to wash and transferred into the new tube. The 50 ml-centrifuge tubes with sediments were then mixed by bubbling with pressurized air for 4 hours. Subsequently, 8 ml of Ultima Gold (Perkin-Elmer, USA) scintillation cocktail was added, and centrifuged at 3500 g for 30 min. The supernatant was transferred into a 20 ml-scintillation vial, and the sediment was resuspended in 8 ml of scintillation cocktail and centrifuged a second time. Radioactivity of sample was measured in a liquid scintillation counter (300SL, Hidex, USA). DCF rates were calculated according to the following formula, which was modified from Molari et al. (2013) [1]:

$$R_{DCF} = \frac{DPM_{inc} \times 1.05 \times DIC \times H}{DPM_t \times V \times t}$$

where DPM_{inc} is the difference between the radioactivities (disintegration per minute; DPM) in the sediment samples and controls; 1.05 is the isotope coefficient used for correcting the uptake of ^{14}C, since the uptake of ^{14}C is 5% lower than the uptake of ^{12}C; DIC is the dissolved inorganic C concentration of overlying water; DPM_t is DPM counts of the added ^{14}C-labeled sodium bicarbonate; V is the volume of sediment; H is the sampling depth; t is the incubation time.

DNA extraction and quantitative PCR (qPCR) analysis

DNA was extracted from 0.25 g of collected sediments using a DNeasy PowerMax Soil Kit (12988-10, QIAGEN, Germany) according to the manufacturer's instructions. Quality assessment was achieved using a NanoPhotometer spectrophotometer (IMPLEN, USA) and a Qubit 2.0 Fluorometer (Life Technologies, USA). qPCR analyses were performed to estimate total prokaryotic microbial abundance of 31 sediment samples. PCR reactions were set up using Bio-Rad SsoAdvanced Universal SYBR Green Supermix under the following conditions: 98°C for 2 min, 30 cycles of 98°C for 30 s, 50°C for 30 s and 72°C for 1 min. Amplification of bacterial and archaeal 16S rRNA genes was performed with the domain-specific primers 338F-806R and 524F10extF-Arch958RmodR, respectively [3]. Standard curves and negative controls for each gene were also conducted. For the determination of gene abundance, only qPCR results exhibiting standard curve correlation coefficients above 0.98, an amplification efficiency exceeding 90%, and a single melting curve peak were selected [4].

16S rRNA gene amplicon sequencing

Microbial community compositions of sediment samples were assessed using high-throughput sequencing of the V3-V4 region of bacterial 16S rRNA genes and the V4-V5 region of archaeal 16S rRNA genes, using the universal primers 338F-806R and 524F10extF-Arch958RmodR, respectively[3]. Polymerase chain reaction products were purified and used to prepare SMRTbell libraries, which were sequenced on the PacBio Sequel platform. The PacBio raw reads were processed for quality and length, and sequences were analyzed using the DADA2 algorithm for identifying mutations and substitutions [5]. The final sequences (amplicon sequence variants, ASVs) were classified using the RDP Classifier against the Silva 16S rRNA database (v138), with a 70% confidence threshold [6].

Metagenomic sequencing, assembly and binning

The metagenomic DNA of thirty-one sediment samples was extracted using the DNeasy PowerMax Soil Kit as described above. DNA library was prepared with

NEBNext UltraTM DNA Library Prep Kit (E7645, NEB, USA) following the manufacturer's protocols. The libraries were then measured using an Agilent 5300 Bioanalyzer (Agilent Technologies, CA, USA) and quantification was done using real-time PCR. Cluster generation was performed on a cBot Cluster Generation System, and paired-end read sequencing (2×150 bp) was performed on the HiSeq 2500 platform (Illumina), generating a ~50 Gb metagenomic dataset per sample. Raw paired-end reads were filtered, quality controlled and trimmed using fastp v0.23.2 with default parameters [7]. All clean reads from different samples were individually assembled with MEGAHIT (v1.1.3) with default settings [8]. Assembled contigs were filtered by length (> 1000 bp) for subsequent binning. Each metagenomic assembly was binned using the metaWRAP v1.3.2 binning module (parameters: -maxbin2 -concoct -metabat2) [9]. All individual assemblies were also concatenated and binned separately using the VAMB tool (v3.0.2; parameters: --minfasta 200000 -o C)[10]. The produced bins from each binning tool were integrated and refined using the Bin_refinement module of metaWRAP (v1.3.2; parameters: -c 50 -x 10). All produced bins were aggregated and dereplicated to a non-redundant set of species-level metagenome-assembled genomes (MAGs) using dRep v3.4.0 (parameters: -comp 50 -con 10) [11] at 95% average nucleotide identities. Completeness, contamination, and heterogeneity of MAGs were evaluated using CheckM v1.1.3 [12]. Taxonomy assignment of each MAG was performed using GTDB-Tk v2.4.0 [13] with reference to GTDB R220 and then validated using a maximum-likelihood phylogenomic tree. The phylogenomic tree based on concatenation of 43 conserved single-copy genes extracted by CheckM v1.2.1 was built using RAxML v8 [14] with the PROTCATLG model.

Metatranscriptomic analysis

Total RNA was extracted from the same samples used for metagenome analysis using the RNeasy PowerSoil Total RNA kit (12866-25, QIAGEN, Germany), according to the manufacturer's instructions. RNA purity and concentration were evaluated using a Qubit 2.0 Fluorometer (Life Technologies, CA, USA). RNA integrity was determined

using an Agilent 5300 Bioanalyzer (Agilent Technologies, CA, USA). Whole transcriptome amplification of total RNA was carried out using the RNA REPLI-g Cell WGA & WTA Kit (150054c, QIAGEN, Germany), according to the manufacturer's protocol. To enrich messenger RNA (mRNA), ribosomal RNA was depleted from total RNA using the RiboCop rRNA Depletion kit (Lexogen, USA). Whole mRNAseq libraries were generated by Majorbio Biotechnology Co. Ltd. (Shanghai, China) using the NEBNext Ultra Nondirectional RNA Library Prep Kit (New England Biolabs), following the manufacturer's recommendations. The constructed libraries were sequenced on a NovaSeq 6000 platform (Illumina) and 150 bp paired-end reads were generated.

Raw metatranscriptomic reads were quality filtered in the same manner as metagenomes. The reads corresponding to ribosomal RNAs were removed using SortMeRNA v.4.3.41 [15] with default parameters with the smr_v4.3_default_db database. Subsequently, these high-quality metatranscriptomic reads were mapped to predicted protein-coding genes from the reference gene catalog and carbon-fixing MAGs using Salmon v.1.9.0 [16] in mapping-based mode (parameters: -validate Mappings -meta). The expression level of each gene was normalized to transcript per million (TPM), based on the gene length and sequencing depth.

Supplementary Figures

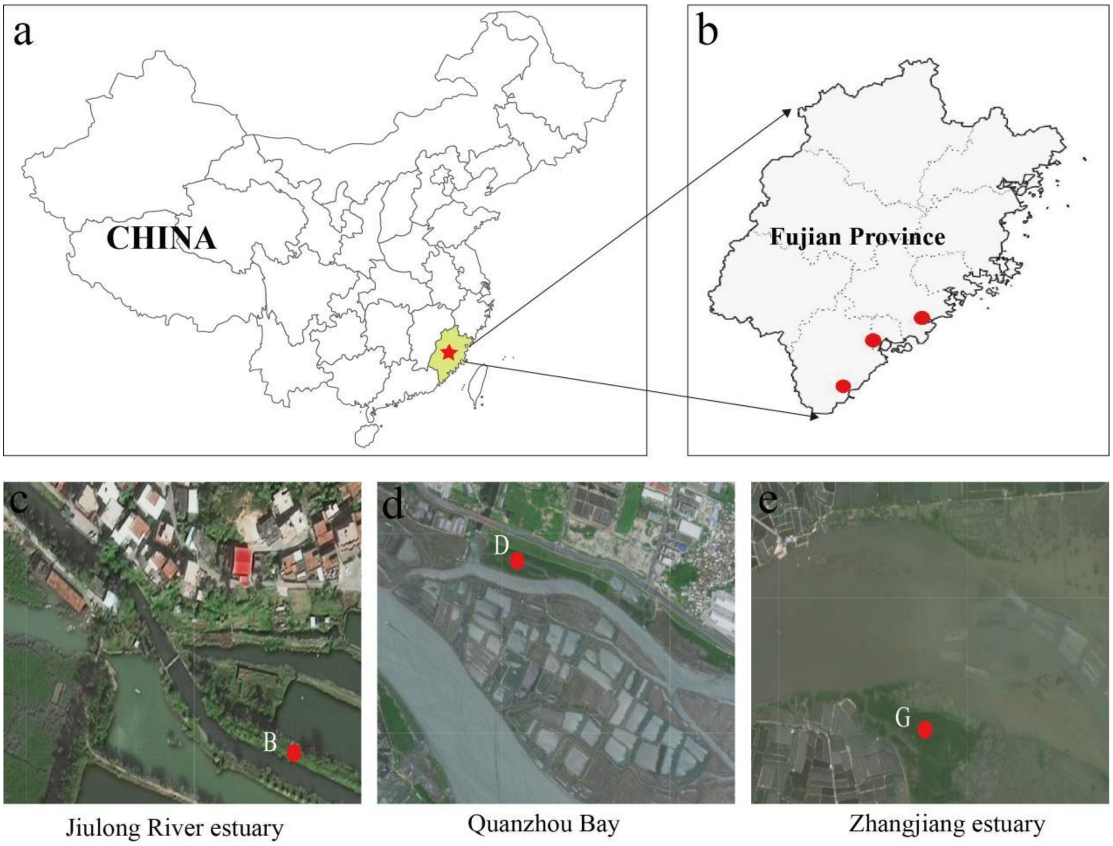

Fig. S1 Location of the sampling habitats at three mangrove wetlands of Fujian Province, China, including Jiulong River estuary, Quanzhou Bay and Zhangjiang estuary.

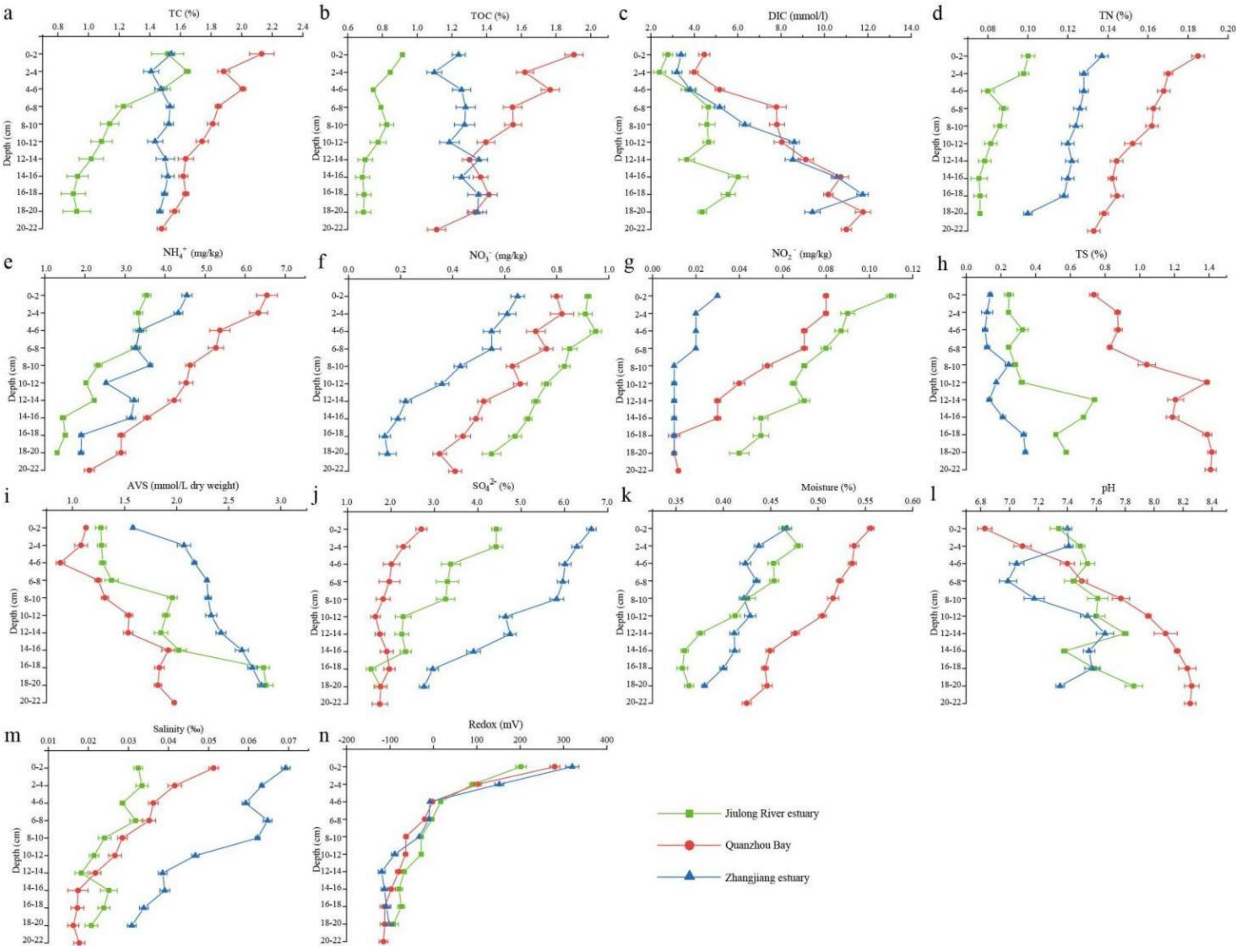

Fig. S2 Vertical distribution of various physicochemical characteristics across the depths of mangrove sediments. Data include the concentrations of TC (a), TOC (b), DIC (c), TN (d), NO_3^- (e), NO_2^- (f), NH_4^+ (g), TS (h), AVS (i), SO_4^{2-} (j), moisture (k), pH (l), salinity (m) and redox (n). Error bars represent standard deviation.

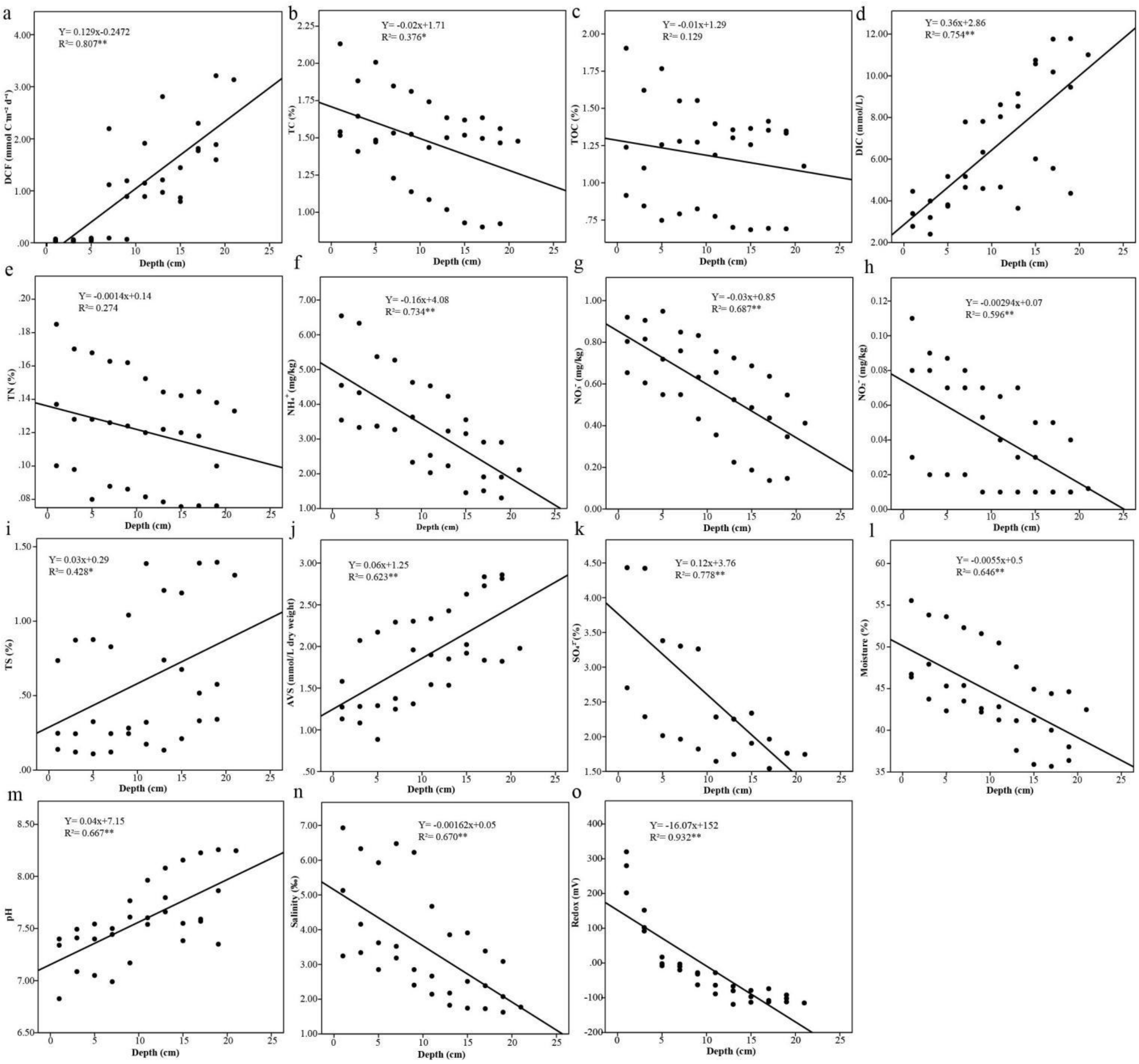

Fig. S3 Curve regression analysis of DCF rates (a) and sediments properties TC (b), TOC (c), DIC (d), TN (e), NO_3^- (f), NO_2^- (g), NH_4^+ (h), TS (i), AVS (j), SO_4^{2-} (k), moisture (l), pH (m), salinity (n) and redox (o) and depths. R^2 was obtained by linear regression analysis and significance levels are denoted with * ($0.01 < P < 0.05$), ** ($0.001 < P < 0.01$) and *** ($P < 0.001$).

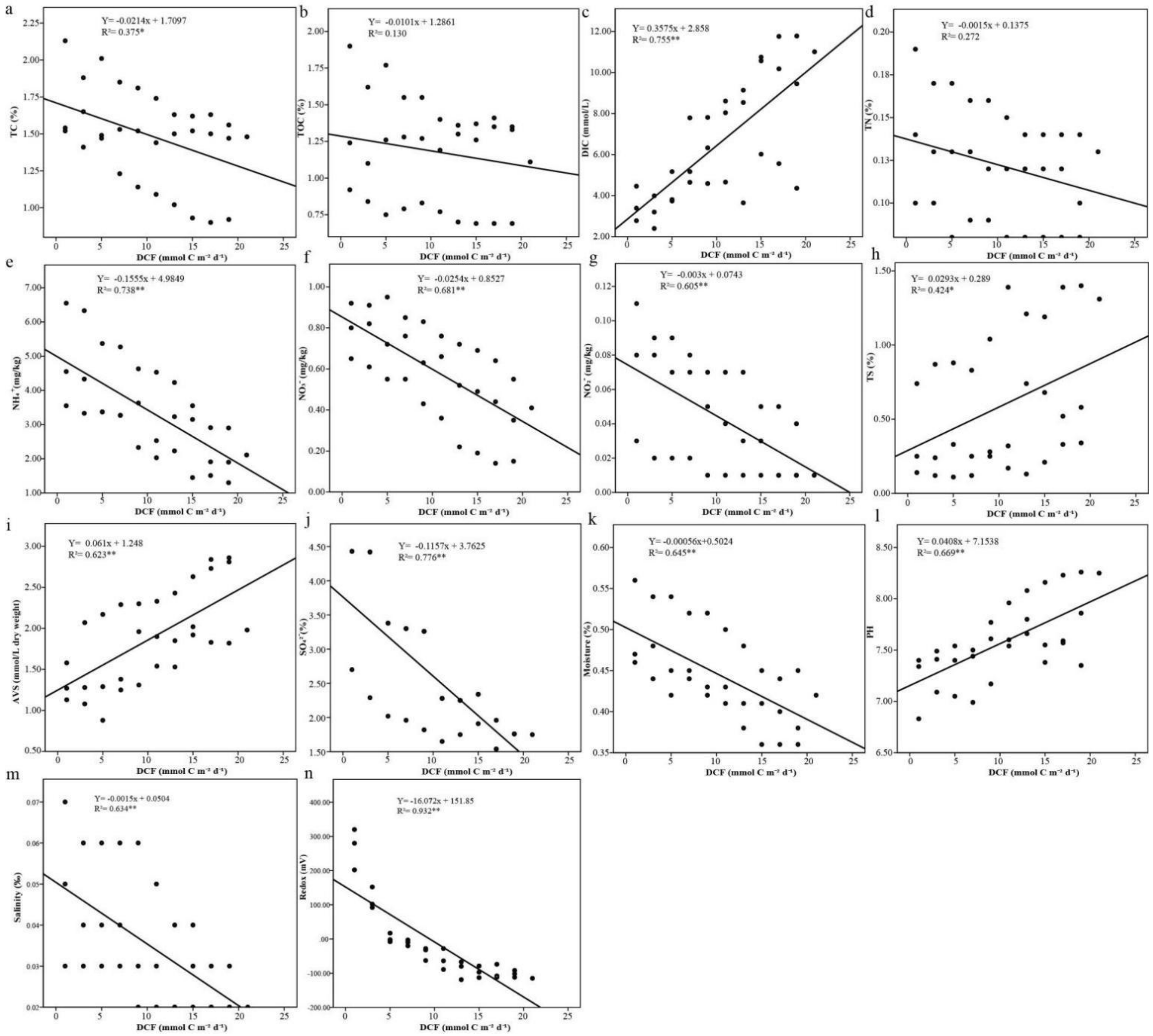

Fig. S4 Curve regression analysis of DCF rates (a) and environmental properties TC (a), TOC (b), DIC (c), TN (d), NO_3^- (e), NO_2^- (f), NH_4^+ (g), TS (h), AVS (i), SO_4^{2-} (j), moisture (k), pH (l), salinity (m) and redox (n). R^2 was obtained by linear regression analysis and significance levels are denoted with * ($0.01 < P < 0.05$), ** ($0.001 < P < 0.01$) and *** ($P < 0.001$).

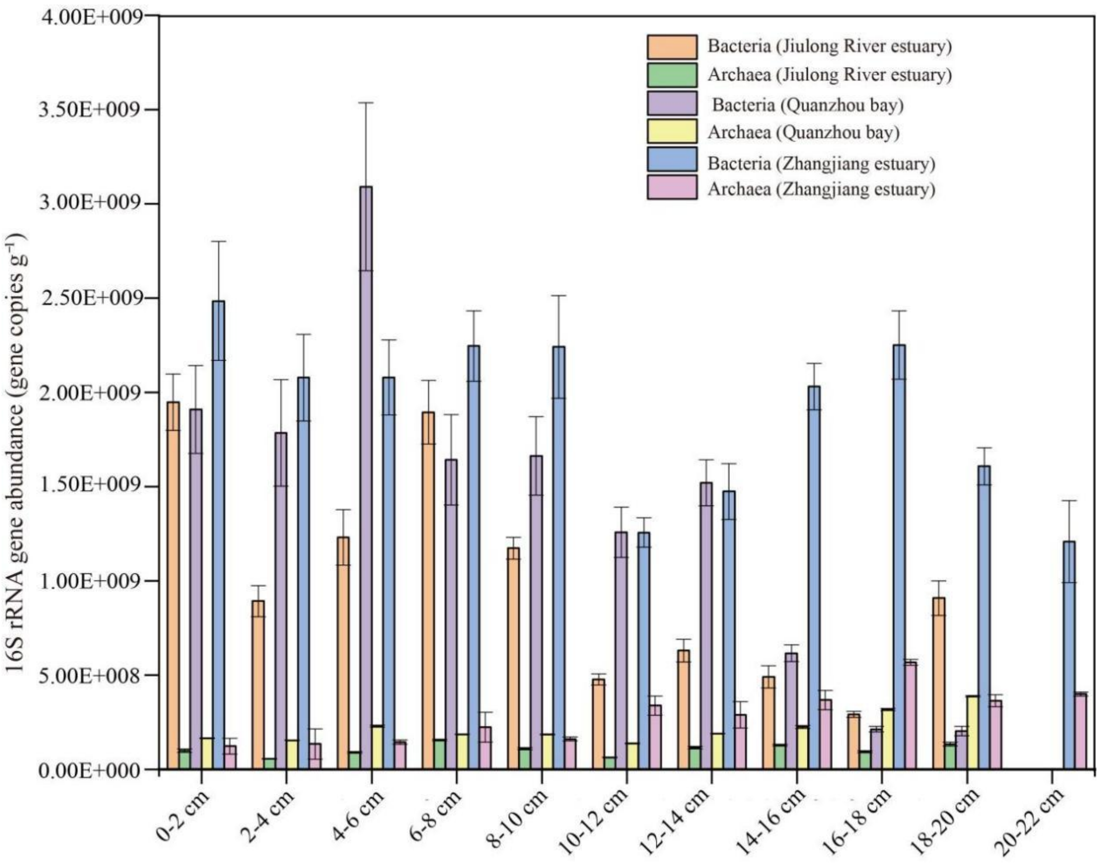

Fig. S5 Quantitative results of the abundance of bacterial and archaeal 16S rRNA gene from 31 mangrove sediment samples of all three sites. Standard deviations are indicated by error bars.

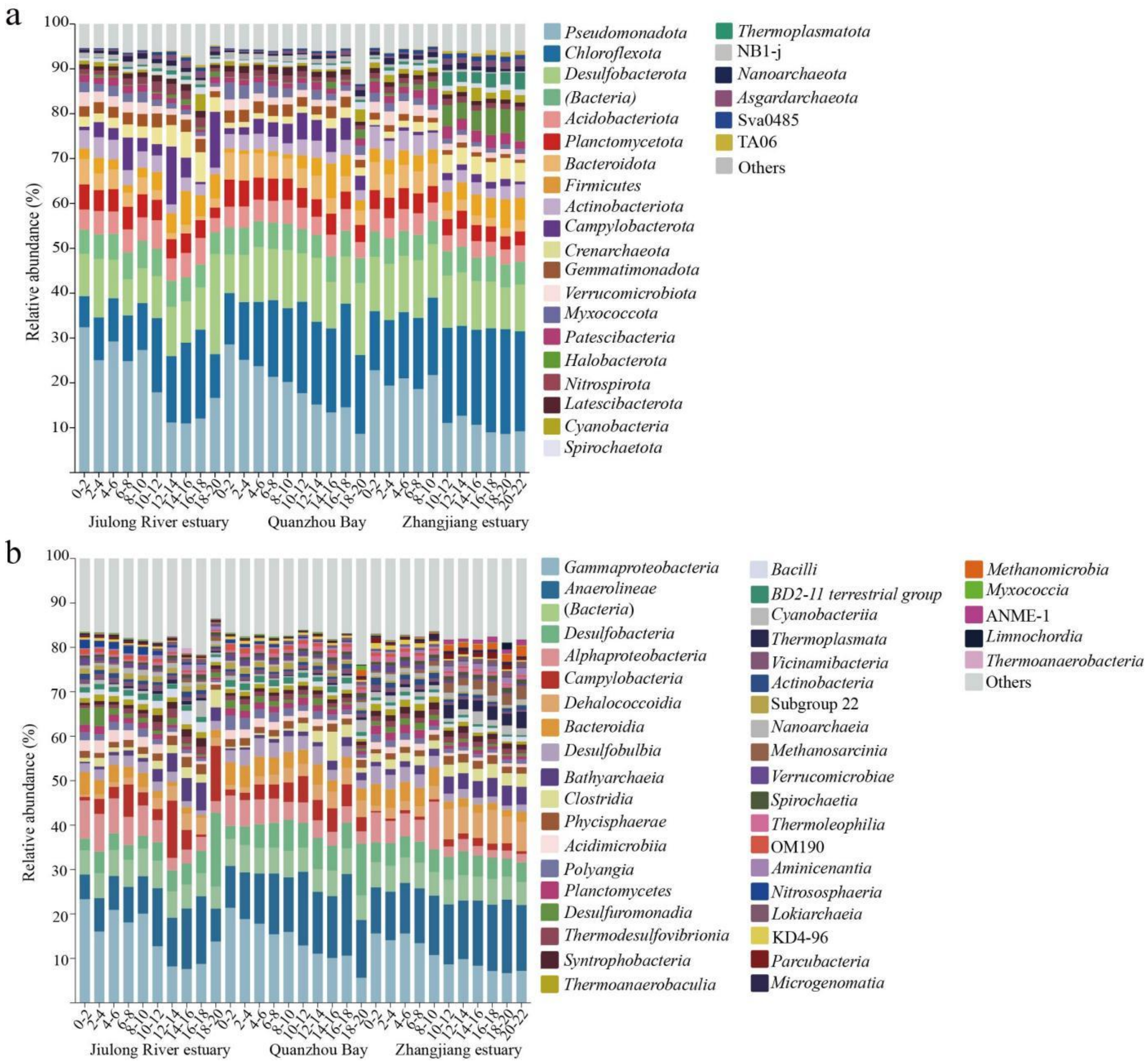

Fig. S6 Taxonomic composition of microbial communities based on 16S miTags extracted from the metagenomes at the phylum (a) and class (b) level. For each plot, all taxa present in a relative abundance of ≥ 1.0% in at least one of the samples are shown, whereas those present in < 1.0% relative abundance are grouped with unassigned sequences in the “Others” category.

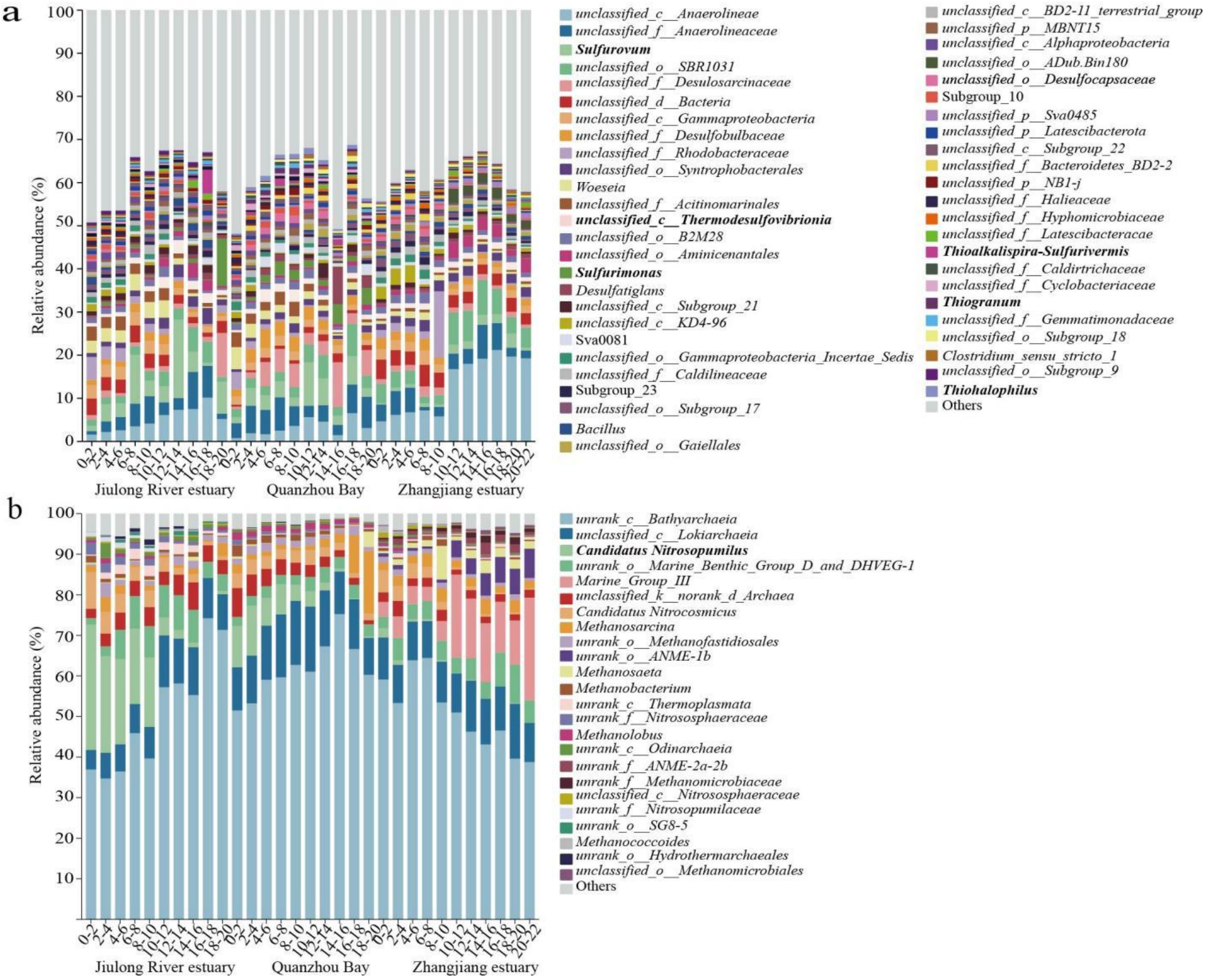

Fig. S7 Taxonomic distribution produced by bacteria (a) and archaeal (b) 16S rRNA gene amplicon sequencing among the 31 mangrove sediments at the genus level. For each plot, all taxa present in a relative abundance of ≥ 1.0% in at least one of the samples are shown, whereas those present in < 1.0% relative abundance are grouped with unassigned sequences in the "Others" category. For plot (a), only the top 50 genera in all samples are presented. The bold fonts represent the conventional chemolithoautotrophic taxa.

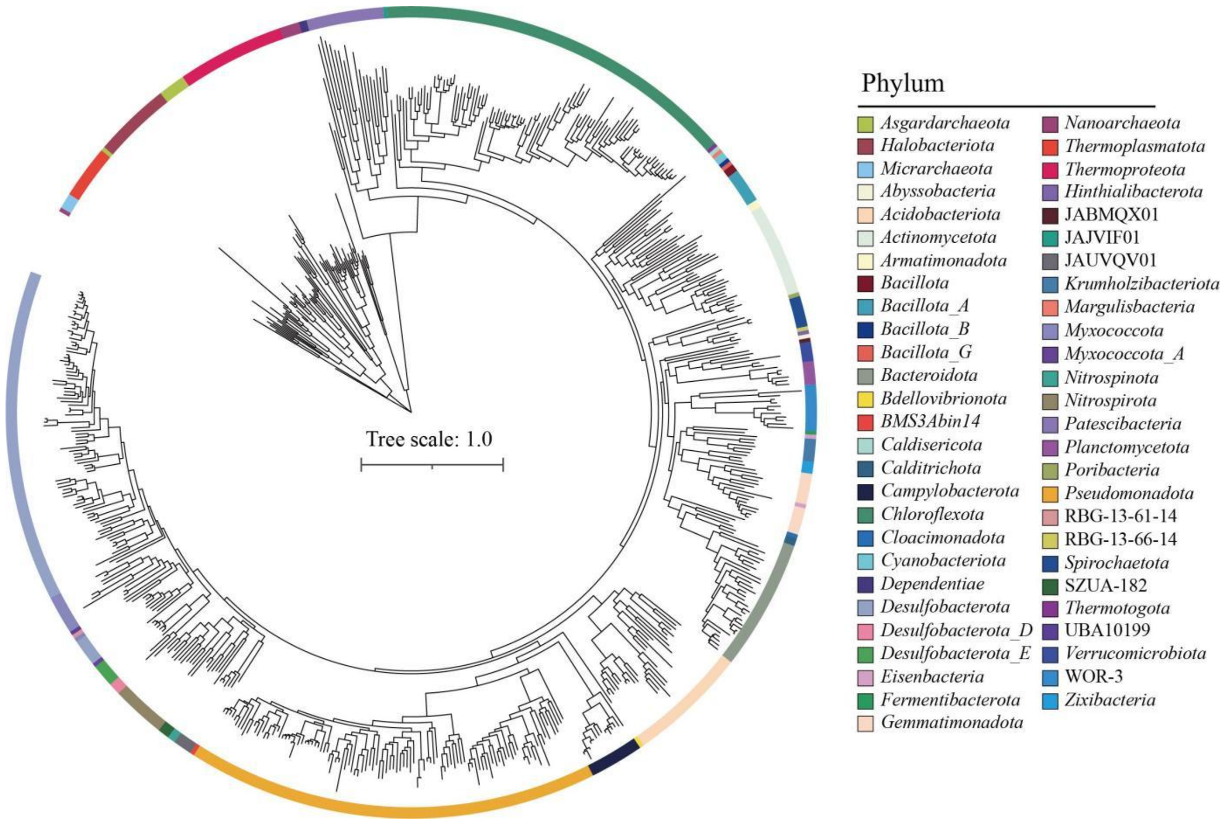

Fig. S8 Phylogenetic placement of 635 MAGs for microbial communities in the mangrove sediments. The maximum-likelihood phylogenomic tree was built based on concatenated amino acid sequences of 43 conserved single-copy genes using RAxML with the PROTCATLG model. The scale bar represents the average number of substitutions per site.

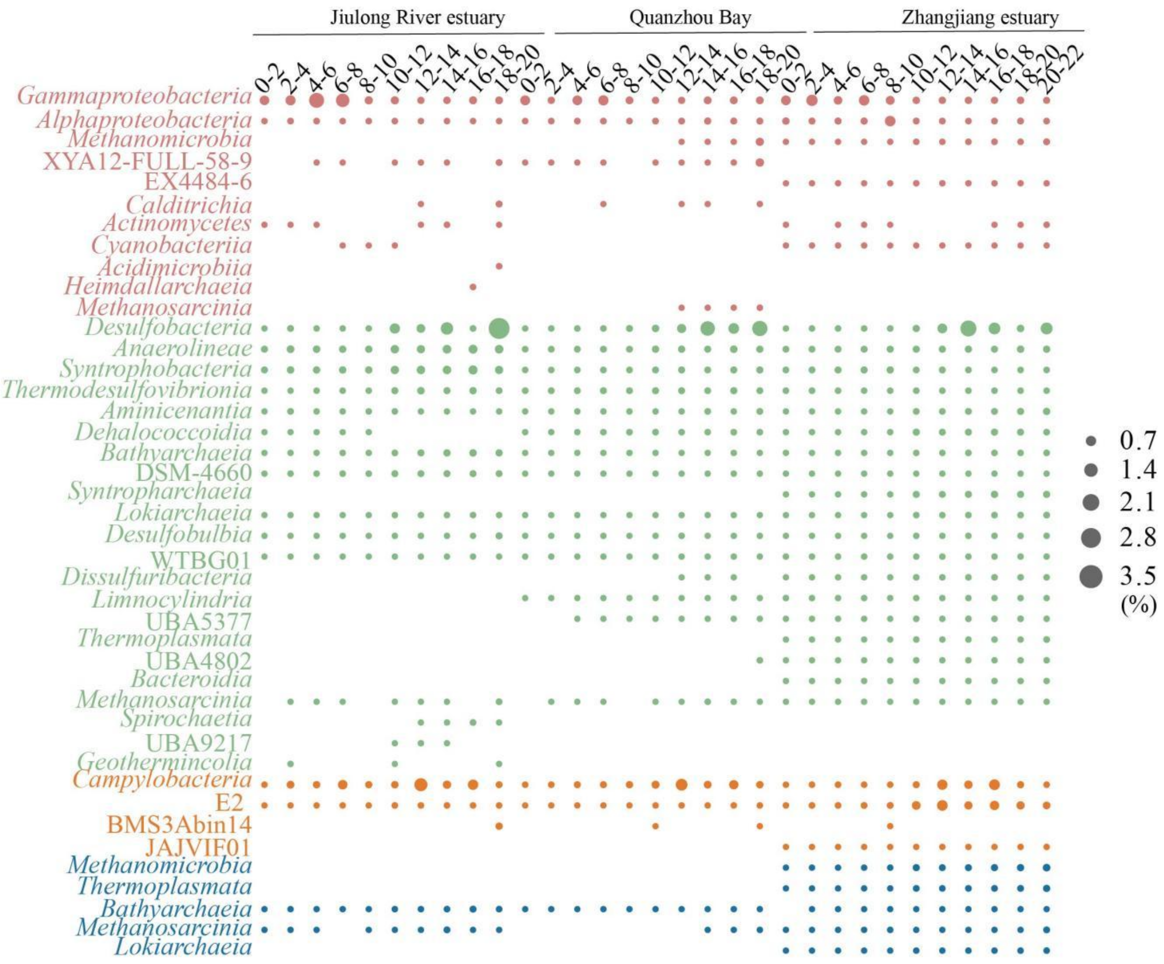

Fig. S9 Relative abundances of carbon-fixing MAGs at the class level in different sediment depths of all three mangrove sites. The circle sizes are proportional to the relative abundance of the sequences per metagenome, and the circle color denotes various carbon fixation pathways.

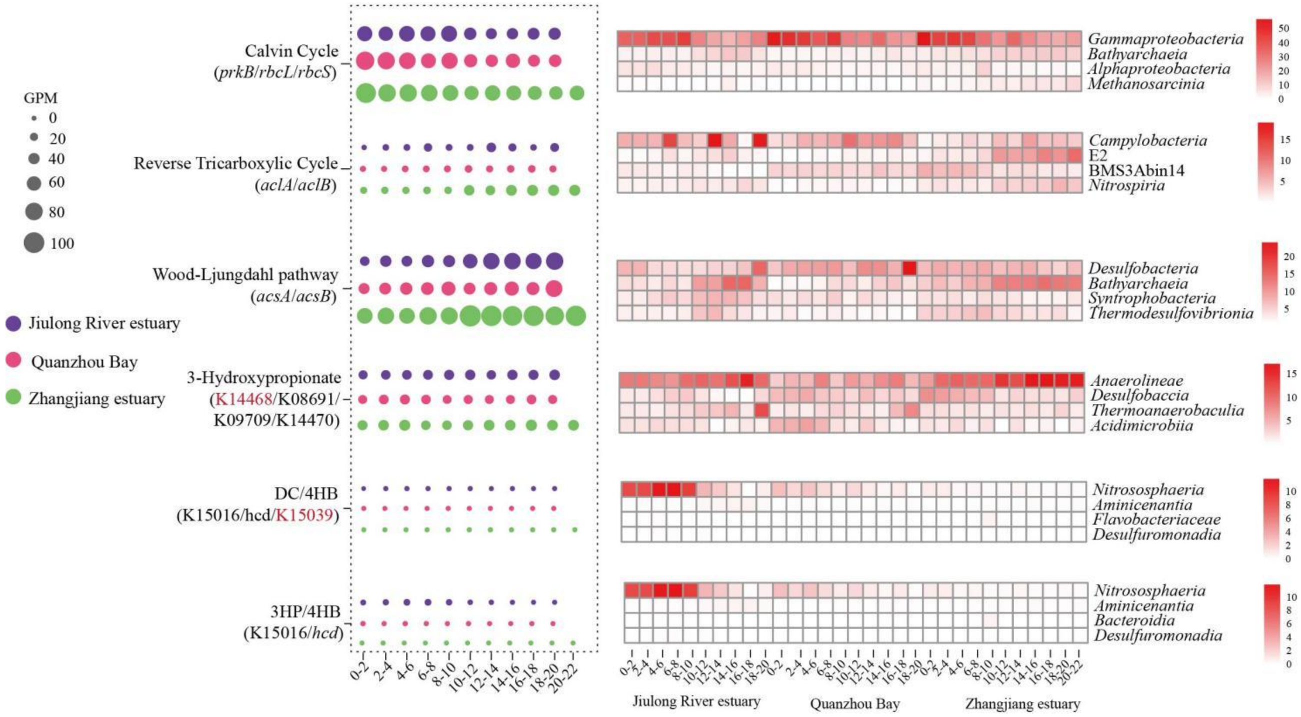

Fig. S10 The distribution of carbon fixation pathways with depth in three mangrove sites. The average abundance of the carbon fixation marker genes at different depths (left). The orange marked genes mean deletion in all samples. The top four affiliated groups containing them shown in the heat map (right) in depth-profiled samples determined by metagenomic analysis.

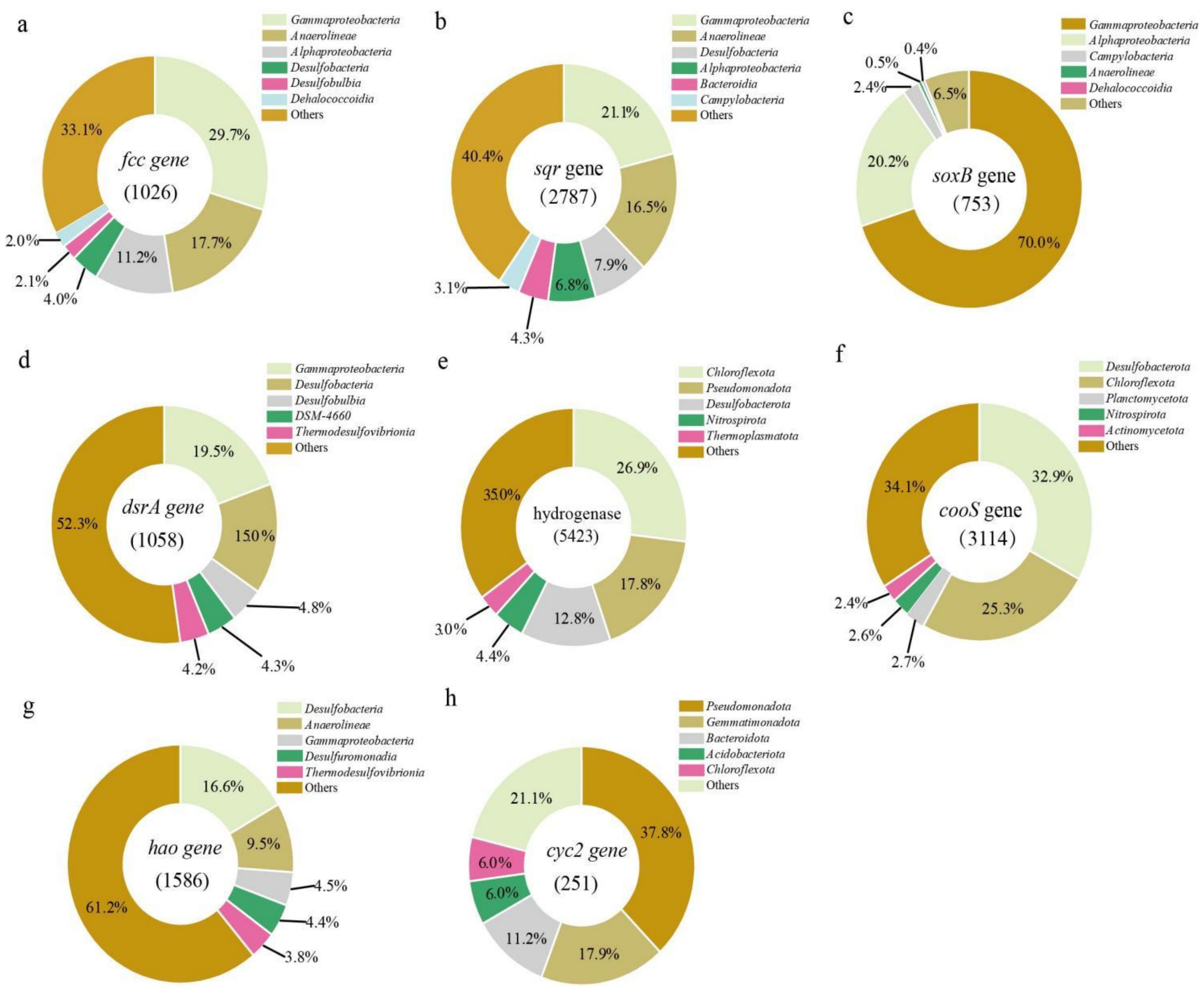

Fig. S11 The proportion of top-ranked microbial taxa involved in different function genes including sulfur oxidation (*fccA*, *sqr*, *soxB*), sulfate reduction (*dsrA*), hydrogenase (*hya*), CO dehydrogenase (*cooS*), hydroxylamine dehydrogenase (*hao*) and iron oxidation (*cyc2*) in the non-redundant gene clusters. The numbers in parentheses indicate sequence counts. Taxonomic annotation was performed with the easy-taxonomy workflow in mmseqs2 (v13.45111) against the Genome Taxonomy Database (GTDB).

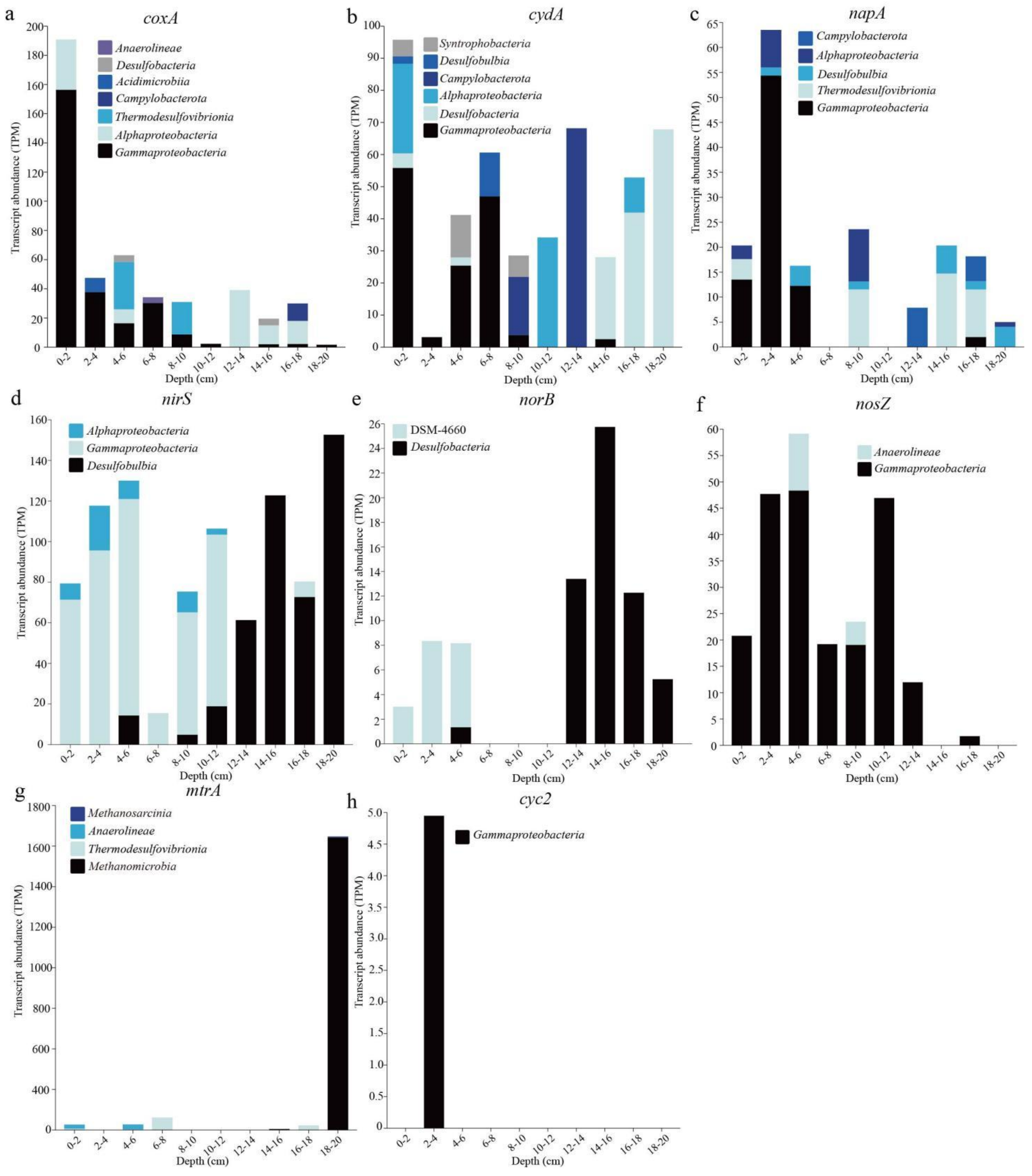

Fig. S12 Transcription of genes of core metabolisms in all carbon-fixing MAGs at different sediment depths, including *coxA* (a), *cydA* (b), *napA* (c), *nirS* (d), *norB* (e), *nosZ* (f), *mtrA* (g) and *cyc2* (h). The expression levels of each gene are represented in units of transcripts per million (TPM).

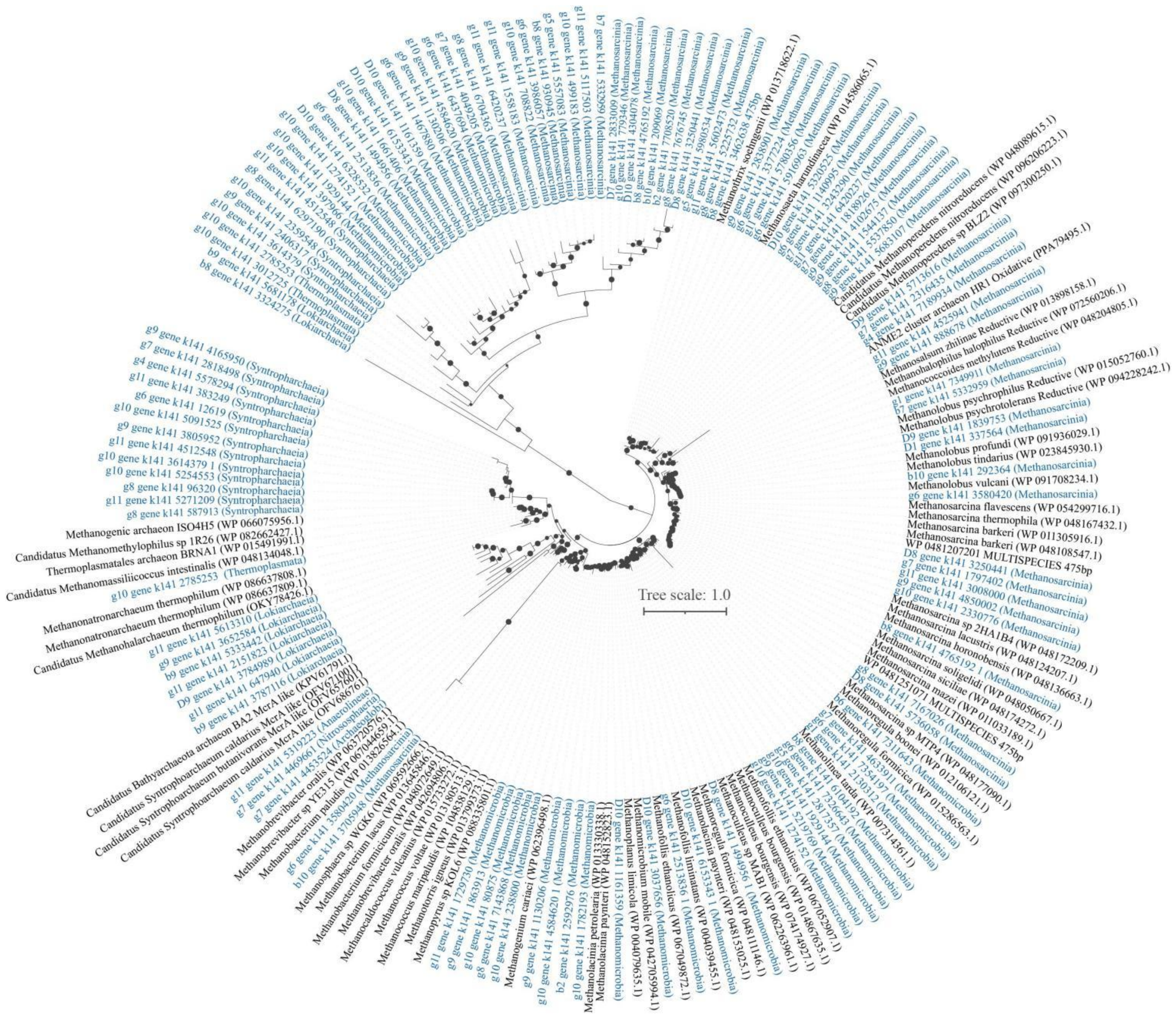

Fig. S13 Maximum-likelihood phylogenetic tree of *mcrA* protein sequence recovered from non-redundant gene catalog. The tree shows sequences from mangrove sediments obtained in this study (blue) alongside representative reference sequences (black). Scale bar indicates the mean number of substitutions per site. Bootstrap values over 50% were shown next to the nodes in phylogenetic trees.

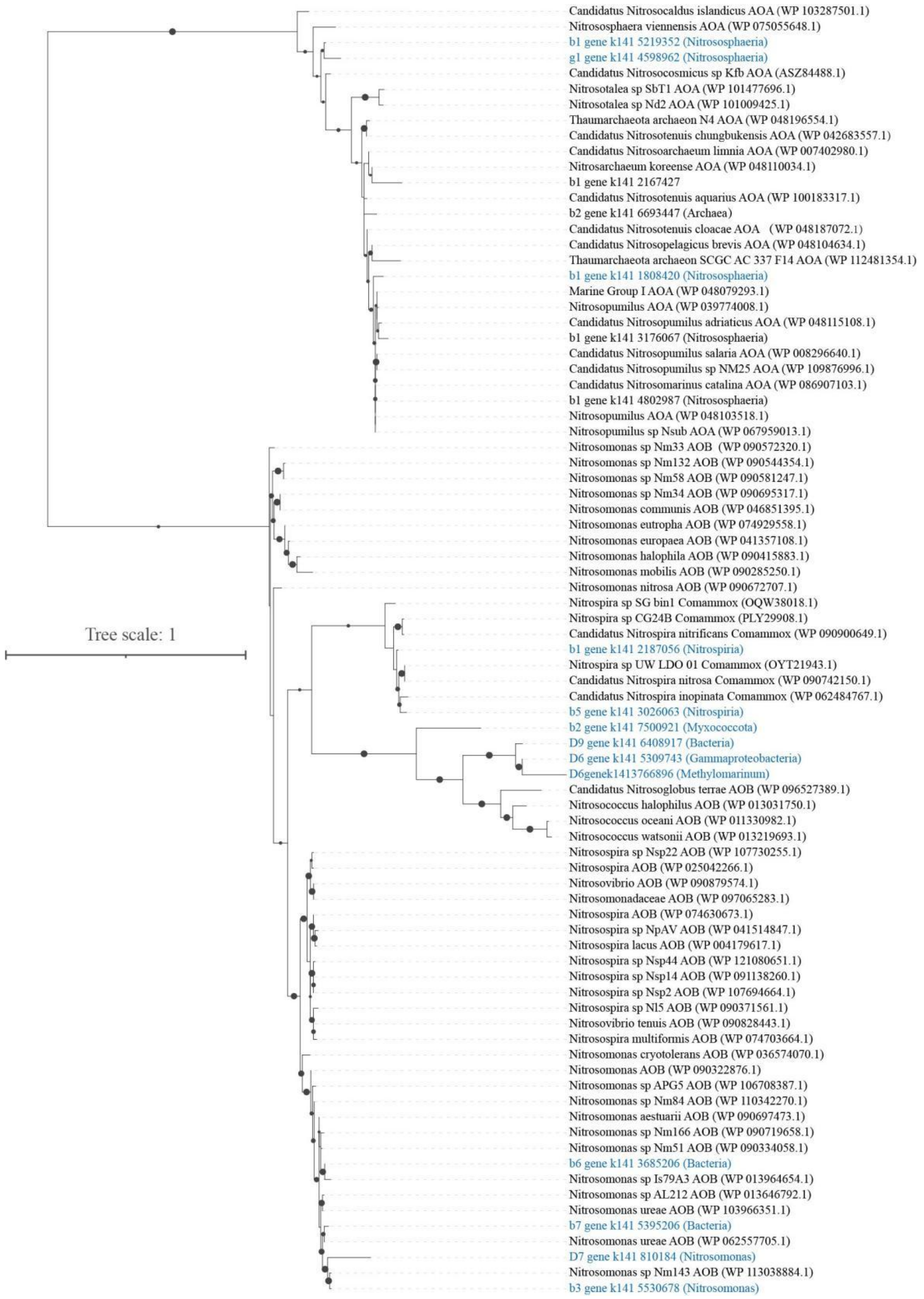

Fig. S14 Maximum-likelihood phylogenetic trees of *amoA* protein sequence recovered from non-redundant gene catalog. The tree shows sequences from mangrove sediments obtained in this study (blue) alongside representative reference sequences (black). Scale bar indicates the mean number of substitutions per site. Bootstrap values over 50% were shown next to the nodes in phylogenetic trees.

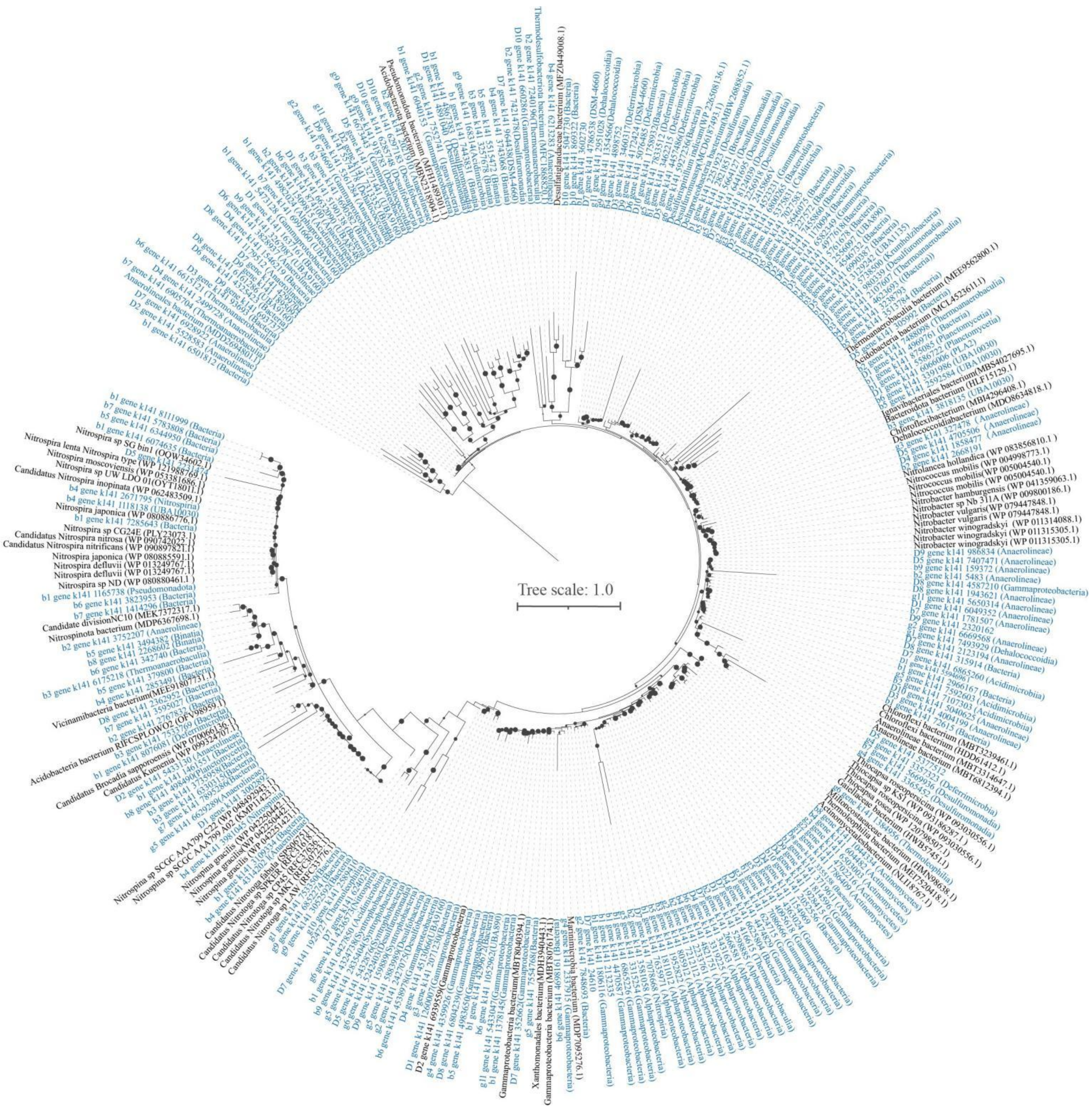

Fig. S15 Maximum-likelihood phylogenetic tree of *nxrA* protein sequence recovered from non-redundant gene catalog. The tree shows sequences from mangrove sediments obtained in this study (blue) alongside representative reference sequences (black). Scale bar indicates the mean number of substitutions per site. Bootstrap values over 50% were shown next to the nodes in phylogenetic trees.

Supplementary references

1. Molari M, Manini E, Dell'Anno A. Dark inorganic carbon fixation sustains the functioning of benthic deep-sea ecosystems. Glob Biogeochem Cy. 2013;27:212-21.
2. Vonnahme TR, Molari M, Janssen F, Wenzhöfer F, Haeckel M, Titschack J, et al. Effects of a deep-sea mining experiment on seafloor microbial communities and functions after 26 years. Sci Adv. 2020;6(18):eaaz5922.
3. Zhou Z, Meng H, Liu Y, Gu JD, Li M. Stratified Bacterial and Archaeal Community in Mangrove and Intertidal Wetland Mudflats Revealed by High Throughput 16S rRNA Gene Sequencing. Front Microbiol. 2017;8:2148.
4. Sun, X. et al. Chemolithoautotropic diazotrophy dominates the nitrogen fixation process in mine tailings. *Environ. Sci. Technol.* **54**, 6082-6093 (2020).
5. Callahan BJ, McMurdie PJ, Rosen MJ, Han AW, Johnson AJ, Holmes SP. DADA2: High-resolution sample inference from Illumina amplicon data. Nat Methods. 2016;13(7):581-3.
6. Henderson G, Yilmaz P, Kumar S, Forster RJ, Kelly WJ, Leahy SC, et al. Improved taxonomic assignment of rumen bacterial 16S rRNA sequences using a revised SILVA taxonomic framework. PeerJ. 2019;7:e6496.
7. Chen S, Zhou Y, Chen Y, Gu J. fastp: an ultra-fast all-in-one FASTQ preprocessor. Bioinformatics. 2018;34(17):i884-90.
8. Li D, Liu CM, Luo R, Sadakane K, Lam TW. MEGAHIT: an ultra-fast single-node solution for large and complex metagenomics assembly via succinct de Bruijn graph. Bioinformatics. 2015;31(10):1674-6.
9. Uritskiy GV, DiRuggiero J, Taylor J. MetaWRAP-a flexible pipeline for genome-resolved metagenomic data analysis. Microbiome. 2018;6(1):158.
10. Nissen JN, Johansen J, Allesøe RL, Sønderby CK, Armenteros JJA, Grønbech CH, et al. Improved metagenome binning and assembly using deep variational autoencoders. Nat Biotechnol. 2021;39(5):555-60.
11. Olm MR, Brown CT, Brooks B, Banfield JF. dRep: a tool for fast and accurate genomic comparisons that enables improved genome recovery from metagenomes

through de-replication. ISME J. 2017;11(12):2864-8.

12. Parks DH, Imelfort M, Skennerton CT, Hugenholtz P, Tyson GW. CheckM: assessing the quality of microbial genomes recovered from isolates, single cells, and metagenomes. Genome Res. 2015;25(7):1043-55.

13. Parks DH, Chuvochina M, Rinke C, Mussig AJ, Chaumeil PA, Hugenholtz P. GTDB: an ongoing census of bacterial and archaeal diversity through a phylogenetically consistent, rank normalized and complete genome-based taxonomy. Nucleic Acids Res. 2022;50(D1):D785-94.

14. Stamatakis A. RAxML version 8: a tool for phylogenetic analysis and post-analysis of large phylogenies. Bioinformatics. 2014;30(9):1312-3.

15. Kopylova E, Noé L, Touzet H. SortMeRNA: fast and accurate filtering of ribosomal RNAs in metatranscriptomic data. Bioinformatics. 2012;28(24):3211-7.

16. Patro R, Duggal G, Love MI, Irizarry RA, Kingsford C. Salmon provides fast and bias-aware quantification of transcript expression. Nat Methods. 2017;14(4):417-9.
